# Decoding the Snail transcriptional network: its role in cancer progression and therapy

**DOI:** 10.1186/s13062-026-00751-1

**Published:** 2026-05-18

**Authors:** S. E. Parfenyev, A. N. Nazarov, A. A. Daks, O. A. Fedorova, N. A. Barlev, O. Y. Shuvalov

**Affiliations:** 1 Institute of Cytology, Russian Academy of Sciences, St. Petersburg, 194064 Russia; 2 Department of Biomedical Sciences, School of Medicine, Nazarbayev University, Astana, 010000 Kazakhstan; 3 National Laboratory of Astana, Astana, 010000 Kazakhstan

**Keywords:** Snail, Epithelial-mesenchymal transition (EMT), E-box, Cancer stem cells (CSCs), EGR1/SP1, Therapy resistance

## Abstract

The transcription factor Snail is a central regulatory hub that governs the transition from localized tumorigenesis to invasive, metastatic, therapy-resistant disease. Elucidating the mechanisms of Snail-driven epithelial-mesenchymal transition (EMT) and identifying strategies to target this pathway are critical challenges and promising frontiers for novel oncology therapeutics. In this review, we systematically analyzed the association between Snail expression and patient outcomes across multiple malignancies based on bioinformatics and statistical interrogation of clinical datasets and molecular interaction networks. Our findings indicate that Snail primarily exerts its oncogenic effects by directly activating a network of pro-metastatic and pro-survival oncogenes, rather than by repressing epithelial genes. We further show that the canonical E-box motif (CANNTG) is a poor predictor of Snail targets. Instead, Snail’s tumor-promoting activity is largely mediated through its cooperation with EGR1/SP1 transcription factors on non-canonical TCACA promoter elements, which upregulate genes such as *ZEB1*, *MMP9*, and *LEF1*. Based on these conclusions, we propose a refined model for predicting Snail target genes. Finally, given that inhibiting the Snail-EMT axis presents a plausible opportunity to limit cancer progression and improve patient outcomes, we also discuss clinically relevant pharmacological strategies for targeting Snail.

**Supplementary Information:**

The online version contains supplementary material available at 10.1186/s13062-026-00751-1.

## Background

The global burden of cancer requires continuous development of new therapeutic strategies that target various biological processes exploited by cancer cells. From this perspective, epithelial-mesenchymal transition (EMT) that plays a pivotal role in tissue and organ formation during embryogenesis, represents a promising therapeutic target. EMT is a fundamental cellular program in which epithelial cells lose apical-basal polarity and cell-to-cell adhesion and adopt a mesenchymal phenotype, concomitantly gaining migratory and invasive properties. In malignancy, tumor cells hijack EMT to drive local invasion, intravasation into blood vessels, and metastasis to distant organs. Moreover, EMT also confers critical tumorigenic properties beyond spreading, that includes therapy resistance, immune evasion, and a stem cell-like state. This makes EMT a central regulator of metastasis-associated cancer lethality.

Snail is a master regulator of EMT [1, 2], alongside other transcription factor families, such as Zeb [3] and Twist [4]. These factors have mutually regulatory relationships that are necessary to ensure the correct and timely execution of this transcriptional program in cells. When aberrantly re-expressed during tumorigenesis, Snail is positively correlated with the aggressiveness of various carcinomas. However, Zeb1 and Zeb2 are more strongly associated with the expression of mesenchymal markers than Twist and Snail [5].

It is important to note that the stability and activity of Snail as a transcription factor in cells critically depend on its post-translational modifications. In this respect, Snail is phosphorylated by GSK-3β, which leads to its ubiquitination and subsequent proteasomal degradation. Conversely, phosphorylation by protein kinase PAK1 stabilizes Snail, significantly extending its lifespan [6]. The acetylation of Snail by the histone acetyltransferases CBP/p300, followed by deacetylation by HDAC1/2, modulates its localization and activity. This regulatory circuitry mediated by histone-modifying enzymes affects both the repressor and activator properties of Snail thereby impacting on the expression of its target genes [7]. It should be taken into account that Snail-specific histone-modifying enzymes operate within multi-protein complexes [7] that are in turn subject to multiple layers of regulation. Therefore, it is not surprising that its activity is modulated by several key oncogenic signaling pathways, including TGF-β–SMAD [8], Akt [9], Notch [10], NF-κB [11], and Wnt [12].

Elucidating the precise mechanisms by which Snail affects downstream pathways and identifying its key effectors in specific cancer types is crucial to our understanding of metastatic mechanisms. In the present manuscript, we combined the literature data with bioinformatic and statistical analysis of publicly available patient data to better understand the prognostic role of the association between *SNAI1* levels, expression of its target genes and patient outcomes in various malignancies. Based on a comprehensive analysis of Snail-binding sites among its * bona fide* target genes verified by ChIP assay, we propose a refined model that may improve the accuracy of predicting Snail targets.

### The role of Snail in oncogenesis at a glance

Snail (*SNAI1*) and Slug (*SNAI2*) are homologous zinc-finger transcription factors belonging to the Snail family of transcription factors. They play an important role in various physiological and pathological processes, from embryonic development to tumor progression. Although Snail and Slug are closely related transcription factors that often work together, they may have distinct, non-redundant functions. For example, *SNAI1* knockout mice are embryonic lethal due to failure in gastrulation, whereas *SNAI2* knockout mice are viable but exhibit defects in neural crest-derived structures [13, 14].

In cancer, Snail is best known as the master regulator of EMT, an important process in the progression of cancer. During EMT, cells increase their migratory and invasive properties, which can lead to metastasis. Metastasis is the leading cause of death in cancer patients [15].

Consistent with Snail’s pivotal role in EMT, p53 and PTEN, critical tumor suppressors in vertebrate cells, counteract its activity [16, 17]. Wild-type p53 was shown to negatively regulate Snail by reducing its protein stability through MDM2-mediated ubiquitination [18]. Furthermore, p53 suppresses Snail expression at the post-transcriptional level by inducing the expression of miR-34a/b/c [19–21]. In contrast, mutant p53 stabilizes Snail and thereby promotes cancer cell invasion [22, 23]. On the opposite, Snail can directly bind to the DNA-binding domain of p53 and hence attenuate its tumor-suppressive activity, which highlights the importance of the reciprocal regulatory loop between p53 and Snail in cancer progression [24].

EMT enables epithelial cells to acquire a mesenchymal phenotype, allowing them to migrate throughout the body and establish secondary tumors. The specific type of EMT depends on the biological context. Type 1 EMT is necessary for proper organ development during embryogenesis. Type 2 EMT is associated with tissue regeneration through fibrosis. Type 3 EMT occurs during tumor development and cancer progression [25].

Snail is one of the main factors that initiate EMT in conjunction with other EMT-associated transcription factors, such as Zeb1, Zeb2, Slug, and Twist. Snail induces EMT through several molecular mechanisms. First, it suppresses the transcription of the *CDH1* gene, which encodes E-cadherin — a molecule crucial for the adhesion of epithelial cells [26]. Snail also downregulates other epithelial markers that play important roles in adhesion, such as desmoplakin, epithelial mucin (MUC1), and keratin 18 [27]. Conversely, Snail positively regulates mesenchymal markers, such as vimentin and fibronectin [28]. These markers also play an important role in EMT. Increasing E-cadherin expression in cells that have undergone a phenotypic change from epithelial to mesenchymal does not revert them to an epithelial morphology [29].

Snail is affected by various extracellular and intracellular stimuli. Thus, many signaling molecules from the tumor microenvironment can upregulate Snail expression, including FGF, TGF-β, BMP, Wnt, PTH(rP), and ILK [30–36]. In addition, several transcription factors, including Lysyl Oxidase Like 2 (LOXL2), Nuclear Factor Kappa B Subunit 1 (NF-κB), Hypoxia Inducible Factor 1 Subunit Alpha (HIF-1α), Inhibitor Of Nuclear Factor Kappa-B Kinase Subunit Alpha (IKKα), SMAD, High Mobility Group AT-Hook 2 (HMGA2), Early Growth Response 1 (Egr-1), Poly(ADP-Ribose) Polymerase 1 (PARP-1), Signal Transducer And Activator Of Transcription 3 (STAT3), Metastasis Associated 1 Family Member 3 (MTA3), and GLI Family Zinc Finger 1 (Gli1), are known to bind to the *SNAI1* promoter, thereby affecting the transcription of the *SNAI1* gene [37]. In turn, Snail transmits received stimuli downstream through its targets, which act as effectors. Early studies suggest that Snail binds to the E-box consensus sequence (CANNTG), thereby regulating gene expression [38]. For example, Snail represses the *CDH1* promoter by binding its C-terminal domain to the 5′–CA(G/C)(G/C)TG–3′ (E-box) elements in the *CDH1* gene promoter that encodes E-cadherin [39]. Similar to the regulation of the *CDH1* gene, Snail acts as a transcriptional repressor of other epithelial genes: desmoplakin *(DSP)* [40], epithelial mucin (*MUC1*) [41], and cytokeratin 18 *(KRT18)* [2]. It performs this function by binding to a DNA site containing the CAGGTG sequence, which is indistinguishable from the E-box of the major binding site of basic helix–loop–helix (bHLH) transcription factors. This creates competition between Snail family proteins and bHLH proteins for the ability to bind to CAGGTG [26, 42].

Thus, Snail is one of the major transcription factors that mediate the EMT program and is affected by various EMT-inducing signals in different types of cancer [43]. This allows cancer cells to invade through the basement membrane into surrounding tissues, intravasate into blood or lymphatic vessels, survive in the bloodstream, and extravasate into distant organs to form metastases.

Beyond metastasis, EMT is closely linked with cancer stem cells (CSCs) and drug resistance [44, 45]. As one of the key EMT drivers, Snail promotes CSC-like properties and confers resistance to therapy [46]. CSCs are a small group of cells within a tumor responsible for initiating and sustaining cancer due to their ability to self-renew and generate diverse tumor cells. Consequently, CSCs are a primary cause of cancer recurrence and metastasis, making them a crucial focus in the development of new therapies. Moreover, CSCs typically overexpress multidrug transporters and have an enhanced capacity for DNA repair. These characteristics ensure their high resistance to chemotherapy and irradiation [1, 47, 48].

For instance, Snail induces resistance to platinum-based drugs, doxorubicin, paclitaxel, temozolamide, gemcitabine, and 5-fluorouracil in different types of malignancies including non-small cell lung cancer [49], ovarian cancer [1], breast cancer [50], head and neck squamous cell carcinoma [51], glioblastoma multiforme [52], colon cancer [53], and pancreatic cancer [54]. In addition, Snail is known to reduce radiosensitivity of cancer cells [55, 56]. For example, it suppresses the transcription of *PTEN* in response to gamma radiation, thereby inhibiting apoptosis [57]. Snail has also been shown to decrease radiosensitivity by arresting the cell cycle in the G2/M phase [58].

Another important role of Snail in cancer is its involvement in the immune evasion of cancer cells. Several molecular mechanisms are known, including Snail-mediated increases in the secretion of C-X-C chemokine ligand 2 (CXCL2), which leads to neutrophil infiltration [59]; recruitment of myeloid-derived suppressor cells (MDSCs) that suppress the immune system [60]; induction of immunosuppressive CD4(+) FOXP3(+) Treg cells [61]; and increased expression of the cytokines CD73, CSF1, and SPP1, which contribute to an immunosuppressive tumor microenvironment [62].

Thus, Snail acts as a multifactorial tumor-promoting factor through various mechanisms, including the initiation of EMT, the promotion of stemness (CSC-like properties), the enhancement of resistance to chemotherapy and radiotherapy, and the modulation of the immune response.

### The role of Snail in various malignancies

As mentioned above, Snail plays an important role in tumor initiation, progression, and drug resistance [63]. Although Snail is mostly associated with negative prognosis for cancer patients, its effect is context-dependent and varies across different cancer types.

**Fig. 1:**
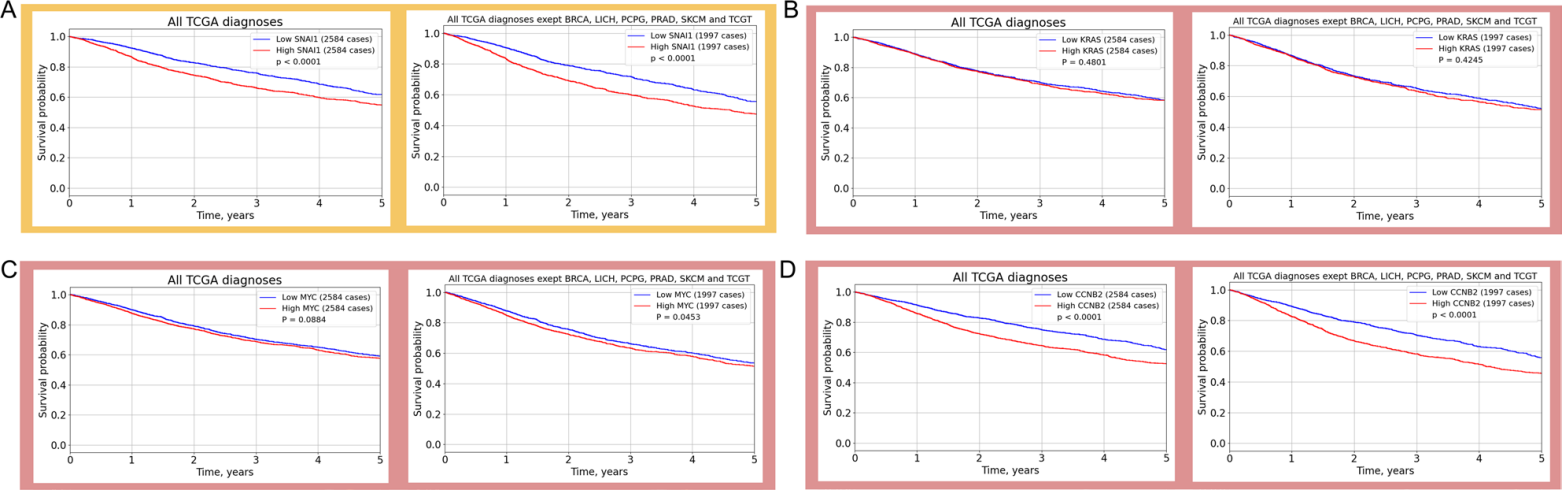
Kaplan-Meier survival plots of cancer patients with high and low levels of *SNAI1* (A), *KRAS* (B), *MYC* (C), and *CCNB2* (D) gene expression (https://portal.gdc.cancer.gov/). Comparisons are made between two groups of patients: those in the upper quartile of gene expression and those in the lower quartile. The survival curves are limited to a five-year time span. The median survival of patients with high and low expression levels of these oncogenes is compared among all patients (left panel) and patients with diagnoses other than BRCA, LIHC, PCPG, PRAD, SKCM, and TGCT (right panel). Excluding the group of patients for whom *SNAI1* does not reduce survival allows us to more clearly observe the effect of* SNAI1* (P < 0.0001 = > P<<0.0001) in the main group of malignancies. A similar effect is observed with the generally recognized oncogenes *MYC* (P = 0.48 = > P = 0.42), *KRAS* (P = 0.09 = > P = 0.05), *CCNB2* (P < 0.0001 = > P<<0.0001)

We analyzed the gene expression and patient overall survival data from The Cancer Genome Atlas (TCGA) project, which is publicly available on the NIH Genomic Data Commons (GDC) Data Portal (https://portal.gdc.cancer.gov/, Data Release 43.0 - May 07, 2025), including about 2,500 patients with 33 types of malignancies. We found that, in general, increased *SNAI1* expression was associated with reduced patient survival across all cancers (Fig. 1A). To better reflect the impact of *SNAI1* on patient survival in the combined cancer cohort we decided to exclude from the analysis a group of malignancies (the “exclusion group”: BRCA, LIHC, PCPG, PRAD, SKCM, and TGCT) in which *SNAI1* expression did not negatively affect survival. This adjustment resulted in a clearer separation of the survival curves depending on *SNAI1* status (Fig. 1). Next, we compared the effect of *SNAI1* with other well-established oncogenes based on the survival curves for patients with high and low expression levels of *MYC*, *KRAS*, and *CCNB2* for both the combined cancer cohort and the cohort lacking the “exclusion group” (Fig. 1). According to these analyses, elevated expression of *SNAI1*, similar to *MYC*, *KRAS*, and *CCNB2*, is negatively associated with patient survival. However, it should be noted that Snail does not consistently worsen the prognosis of cancer patients and may not exhibit oncogenic activity in certain cancer types.

**Fig. 2:**
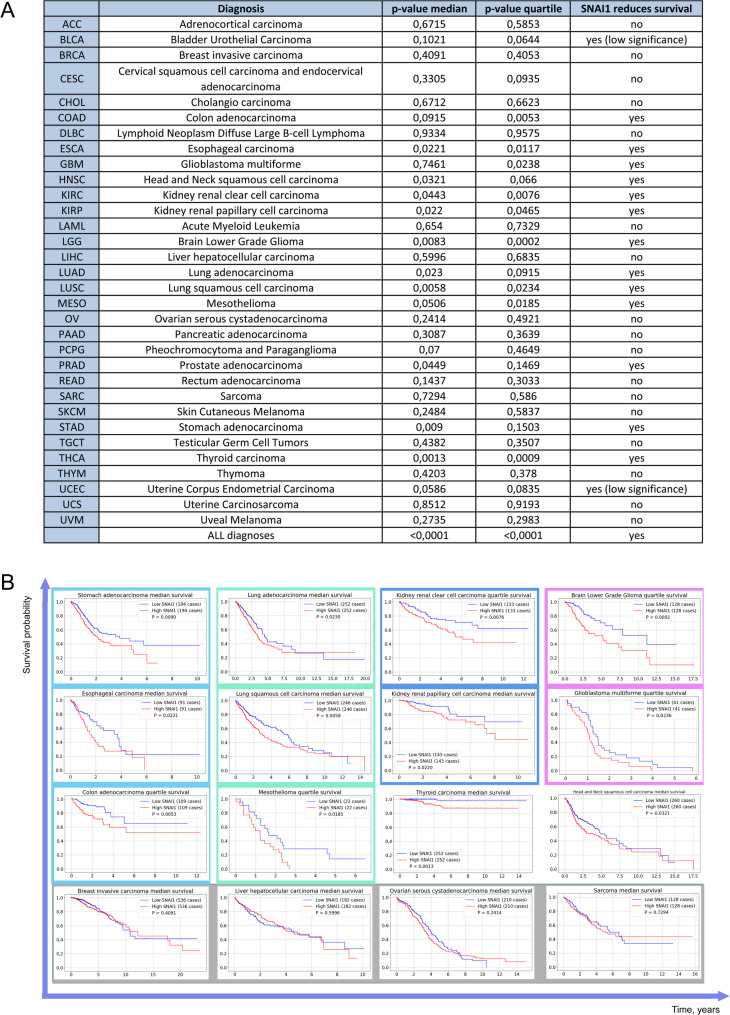
The comparison of life expectancy between cohorts with high and low *SNAI1* expression was carried out using the log-rank test at a significance level of 0.05. (A) The table demonstrates the impact of *SNAI1* expression level on survival probability of cancer patients based on the p-value median or p-value quartile stratification. (B) Survival Kaplan–Meier plots of cancer patients with high and low *SNAI1* expression level

A more detailed survival analysis of these cohorts revealed heterogeneity among different cancer types with respect to *SNAI1* status (Fig. 2). Approximately in a half of malignancies, the patient survival rates inversely correlated with *SNAI1* levels. These types include: colon adenocarcinoma (COAD), esophageal carcinoma (ESCA), glioblastoma multiforme (GBM), head and neck squamous cell carcinoma (HNSCC), kidney renal clear cell carcinoma (KIRC), kidney renal papillary cell carcinoma (KIRP), brain lower grade glioma (LGG), lung adenocarcinoma (LUAD), lung squamous cell carcinoma (LUSC), mesothelioma (MESO), stomach adenocarcinoma (STAD), and thyroid carcinoma (THCA) (Fig. 2). However, in some malignancies, patient survival tends to increase, including bladder urothelial carcinoma (BLCA), cervical squamous cell carcinoma and endocervical adenocarcinoma (CESC), ovarian serous cystadenocarcinoma (OV), rectal adenocarcinoma (READ), and uterine corpus endometrial carcinoma (UCEC) (Fig. 2A). Notwithstanding, *SNAI1* expression did not correlate with survival outcomes in patients with the following malignancies: adrenocortical carcinoma (ACC), breast invasive carcinoma (BRCA), cholangiocarcinoma (CHOL), diffuse large B-cell lymphoma (DLBCL), kidney chromophobe (KICH), acute myeloid leukemia (LAML), liver hepatocellular carcinoma (LIHC), pancreatic adenocarcinoma (PAAD), pheochromocytoma and paraganglioma (PCPG), sarcoma (SARC), skin cutaneous melanoma (SKCM), testicular germ cell tumors (TGCT), thymoma (THYM), uterine carcinosarcoma (UCS), and uveal melanoma (UVM). Finally, an inverse relationship was observed in prostate adenocarcinoma (PRAD): the overexpression of *SNAI1* is associated with an increase in life expectancy (Fig. 2A).

As shown in Fig. 2, *SNAI1* is negatively associated with survival in only half of the malignancy types examined (19 out of 33). Probably, this can be explained by the fact that Snail regulates many targets, the molecular functions of which can differ depending on the complex genetic background, including the type of tissue. Additionally, Snail expression depends on a number of complicated, interconnected signaling pathways.

As shown in Fig. 2A and survival graphs (Fig. 2B), the link between *SNAI1* expression level and patient survival rate is similar among malignancies derived from the related tissues. For example, in malignancies of digestive tract (ESCA, COAD, STAD), kidney (KIRC, KIRP) or lung tumors (LUAD, LUSC, MESO), high expression of *SNAI1* is negatively associated with patient outcome. In the group of the female reproductive system malignancies (OV, CESC, UCEC), there is only a tendency to depend on the level of *SNAI1*. At the same time, in the case of tumors of the accessory digestive organs (LIHC and PAAD), there is no association between survival of cancer patients and the level of *SNAI1*.

#### Cancers of the digestive tract

Cancers of the digestive tract, including those of the esophagus, stomach, and colon, account for the largest number of new cancer cases and deaths each year [64]. In all of these types of cancer, *SNAI1* expression is associated with poorer patient survival (Fig. 2).

There are dozens of examples illustrating the mechanisms underlying the oncogenic role of Snail in esophageal, gastric, and colorectal cancer which are summarized in a number of excellent reviews [2, 65, 66].

For instance, in gastrointestinal cancer, Fas signaling drives metastasis by triggering EMT through an ERK/MAPK pathway that stabilizes Snail and β-catenin via GSK-3β inactivation [67]. In addition, Xu et al. have demonstrated that hyperglycemia promotes tumor progression by upregulating the glycolytic enzyme ENO1, which activates the TGF-β/Smad signaling pathway to drive Snail-induced EMT [68]. In turn, Snail promotes aerobic glycolysis and tumor progression in gastric cancer by directly repressing the expression of the metabolic enzyme FBP1 [69]. Moreover, Snail maintains cancer stem cell-like properties and tumorigenicity in gastric cancer by the upregulation of Cellular Communication Network Factor 3 (CCN3) and downregulation of Neurofilament Light Polypeptide (NEFL) [70].

In colorectal cancer, Snail promotes lung metastasis by driving EMT and stimulating cancer cells to secrete CXCL2, which recruits and activates metastasis-promoting M2 macrophages [71]. Moreover, Snail induces cancer stem cell-like properties by directly activating interleukin-8 (IL-8) expression [72]. IL-8 is a critical chemokine promoting EMT and stemness in colorectal cancer [73]. Additionally, the transcription factor KLF4 maintains stemness and mesenchymal properties in colorectal cancer stem cells by activating the TGF-β1/Smad/Snail signaling pathway [74].

Finally, Snail confers 5-fluorouracil resistance in colorectal cancer by directly binding to the *ABCB1* gene promoter and upregulating the expression of this drug efflux pump [53]. The downregulation of Snail sensitizes esophageal carcinoma to cisplatin [75] and irradiation [76].

#### Lung cancers and mesothelioma

There are two main types of lung cancer: non-small cell lung cancer (NSCLC) and small cell lung cancer (SCLC). These types differ in clinical manifestations and prognosis [77]. The most common types of NSCLC are squamous cell carcinoma, adenocarcinoma, and large cell carcinoma [78]. Analysis of data from the GDC Data Portal (portal.gdc.cancer.gov) reveals a significant negative association between *SNAI1* expression and patient survival in cohorts diagnosed with lung squamous cell carcinoma or adenocarcinoma (Fig. 2). This correlation is consistent with a substantial body of experimental evidence confirming the pro-oncogenic role of Snail in lung malignancies.

The cytokine transforming growth factor beta 1 (TGF-β1) promotes cell invasion, migration, and proliferation in lung cancer through the Smad/Snail signaling pathway [79]. As an effector of the Stat3/Snail/MMP2/MMP9 signaling axis, Snail promotes the migration and invasion of lung cancer cells [80, 81].

Snail has been shown to promote angiogenesis and tumor progression by activating signaling pathways associated with the chemokines CXCL8 and CXCL5 [82]. Snail-induced EMT promotes the delivery of exosomal miR-21 to tumor-associated macrophages. This miRNA inhibits inflammasome assembly, thereby suppressing NLRP3 activation and contributing to the establishment of an immunosuppressive tumor microenvironment [83].

In NSCLC, Snail promotes metastasis and stem cell-like properties by increasing Nanog expression via the hyperactivation of Smad1 and Akt, as well as the inhibition of GSK3β [84]. The Snail expression is upregulated in osimertinib-resistant lung cancer cells, whereas its knockdown increases their sensitivity. Furthermore, the CDK4/6 inhibitor palbociclib and MET inhibitor capmatinib were shown to downregulate Snail and, when combined with osimertinib, could reverse this acquired resistance [49, 85].

Mesothelial cells form the serous membrane of the pleural, pericardial and peritoneal cavities [86]. Pleural mesothelioma, the most common form of this malignancy, is primarily caused by the inhalation of asbestos fibers. These fibers induce chronic inflammation and oncogenic transformation in mesothelial cells [87]. Snail has been shown to directly bind to p53, a critical tumor suppressor, thereby inhibiting its function [88]. In the context of pleural mesothelioma, Schelch et al. demonstrated that the oncoprotein YB-1 promotes aggressive cellular behaviors, including migration and intravasation, in a Snail-dependent manner. This effect is mediated by the precise regulation of epidermal growth factor receptor (EGFR) signaling [89].

#### Renal cell carcinoma

Renal cell carcinoma (RCC) is a type of kidney cancer that arises from the renal epithelium. It accounts for about 90% of kidney cancers and includes more than 10 subtypes, including the most common and lethal types — KIRC and KIRP [90]. The tumor suppressor gene *VHL* is the most frequently mutated gene in renal cell carcinoma (RCC). Its inactivation leads to stabilization of hypoxia-inducible factors (HIFs) and subsequent upregulation of target genes involved in angiogenesis, apoptosis, and glycolysis [90]. A growing body of evidence implicates Snail in RCC pathogenesis. Its expression correlates with invasion, metastasis, and advanced stage [91]. Functionally, Snail drives clear cell RCC (ccRCC) progression through the AKT/STAT3/MAPK cascade [92] and promotes metastasis via the GHSR-PI3K-Akt axis [93]. Consistent with these pro-oncogenic functions, analysis of the GDC Data Portal (https:// portal.gdc.cancer.gov) reveals a significant association between high *SNAI1* expression and poor patient prognosis (Fig. 2A).

#### Gliomas

The most common primary brain tumors in adults are gliomas, which can be classified based on their histological type into the astrocytic, oligodendroglial, or oligoastrocytic lineages. They can also be divided into grades: Grade I (pilocytic astrocytomas), Grade II (low-grade), Grade III (anaplastic), and Grade IV (glioblastoma). The grade depends on the number of malignant signs present [94].

Glioblastoma penetrates deeply into surrounding tissue but rarely metastasizes to other organs [95]. Functional studies have established Snail as a critical driver of glioblastoma pathogenesis. It is required not only for cell migration and invasion but also for cell cycle progression, viability, and proliferation of glioblastoma cells *in vitro *[96, 97]. Consistent with these pleiotropic effects, Snail integrates multiple oncogenic signaling pathways in gliomas. For instance, Liver kinase B1 (LKB1) exerts tumor-suppressive effects by negatively regulating the NF-κB/Snail axis to inhibit proliferation, migration, and invasion [98], whereas the High-Grade Glioma F-Box Protein (FBXO17) enhances tumor aggressiveness through AKT/GSK-3β-dependent Snail activation [99].

The clinical importance of this regulatory network is supported by survival data from the GDC portal (https://portal.gdc.cancer.gov/), where high *SNAI1* expression predicts reduced life expectancy of patients diagnosed with glioblastoma or LGG.

#### Thyroid carcinoma

Thyroid cancer comprises several histological subtypes, most notably follicular and papillary carcinomas, which exhibit distinct expression profiles of genes regulating the cell cycle, apoptosis, and cell adhesion [100]. Among these, *SNAI1* is specifically upregulated in malignant thyroid tissue compared to normal tissue, suggesting that Snail plays a role in thyroid carcinogenesis [100]. Multiple lines of evidence support a pro-oncogenic role for Snail in papillary thyroid carcinoma. Wang et al. demonstrated concurrent upregulation of Snail with other EMT-associated factors, including TGF-β1, Snail, and MMP9, in papillary thyroid carcinoma (PTC). They also found a correlation between the expression of these factors [101]. Another study showed that the BRAF V600E mutation in the B-type Raf kinase gene, a key driver of PTC, promotes tumor cell migration and invasion by inducing EMT through Snail upregulation [102]. Consistent with these findings, analysis of patient survival data reveals that elevated *SNAI1* expression is significantly associated with reduced survival rates in thyroid carcinoma (Fig. 2).

#### Head and neck squamous cell carcinoma (HNSCC)

HNSCC is the most common malignancy arising from the mucosal epithelium of the oral cavity, pharynx, and larynx. It is etiologically linked to carcinogen exposure and inactivation of tumor suppressors such as p53, CDKN2A, and PTEN [103]. The EMT-inducing transcription factors Snail and Twist are frequently activated in this context, often downstream of hypoxia-driven HIF1α induction. Snail has been shown to promote metastasis in HNSCC cells through its involvement in the PI3K/AKT/Snail signaling axis [104].

Long-term exposure to the tobacco carcinogen NNK enhances the malignant progression of HNSCC by increasing cell migration, invasion, and cancer stem cell properties. This aggressive behavior is mediated through the Snail-RKIP signaling pathway, which also confers increased anti-apoptotic ability and therapeutic resistance [105]. Another study noted that Snail increases the invasive and migratory capacity of HNSCC cell lines and enhances their resistance to erlotinib [106]. These data align with the finding that *SNAI1* expression is associated with worse survival in patients diagnosed with HNSCC.

#### Malignancies in which SNAI1 expression is not reliably associated with patient survival

Snail is widely recognized as an oncogenic driver of EMT and metastasis, typically associating with poor prognosis in cancer patients. However, this relationship is not universal; in approximately half of all malignancies, *SNAI1* expression shows no significant correlation with patient survival (Fig. 2).

#### Breast cancer

A recent study has elucidated a critical role for chaperone-mediated autophagy (CMA) in regulating Snail stability and, consequently, Snail-driven EMT in breast cancer cells. The chaperone HSC70 binds Snail and targets it for lysosomal degradation [107]. Importantly, the efficiency of this regulatory mechanism varies significantly among breast cancer subtypes. In luminal-type breast cancer, Snail undergoes CMA-mediated degradation. However, in triple-negative breast cancer (TNBC), Snail evades this process [107]. This differential regulation at the protein level likely explains why there is no significant correlation between *SNAI1 *mRNA expression and patient survival in the BRCA cohort.

#### Hepatocellular carcinoma (HCC)

Using hydrodynamic transfection to deliver a *SNAI1*-containing vector into mouse liver, Xu et al. demonstrated that Snail overexpression alone is insufficient to promote liver tumorigenesis, accelerate the development of AKT/c-Met-induced liver cancer, or drive distant metastases in mice [108]. Notably, E-cadherin expression was present in both normal liver tissue and liver tumors. Combined overexpression of Snail and activation of the Notch and YAP signaling pathways did not increase proliferation [108]. These results suggest that, while Snail is sufficient to trigger EMT in vitro, it requires additional cofactors to exert similar effects during hepatocarcinogenesis in vivo [108].

#### Pancreatic ductal adenocarcinoma (PDAC)

Studies in intestinal and pancreatic models reveal tissue-specific requirements for Snail in EMT and tumorigenesis. Paul et al. demonstrated that Snail overexpression in adenomas and carcinomas fails to suppress E-cadherin, reflecting its inability to initiate a full EMT program [109]. Similarly,* SNAI1* appears dispensable for murine pancreatic ductal adenocarcinoma (PDAC) development [110], with Zeb1 likely serving as the predominant EMT driver in this context [111].

Consistent with its dispensable role in pancreatic tumorigenesis, Snail loss does not promote epithelial differentiation or inhibit the progression of EMT-established PDAC. Instead, Snail contributes to PDAC pathogenesis through alternative mechanisms, including the promotion of chemoresistance, which likely underlies the reduced lifespan of patients with elevated Snail expression [109]. Mechanistically, Snail enables evasion of KRAS-mediated senescence by inducing p16INK4A-dependent cell cycle arrest [109]. Notably, this function reflects a non-canonical role for Snail as a transcriptional activator, rather than its classical function as a repressor of epithelial genes [26].

#### The effect of Snail on cancer patient survival assessed by immunohistochemistry

Ideally, the functional impact of Snail should be assessed at the protein level, because protein abundance and post‑translational modifications ultimately determine its biological activity. However, comprehensive, publicly available datasets linking Snail protein expression to patient survival are currently lacking. Therefore, in the present study, we relied on *SNAI1* mRNA expression data from The Cancer Genome Atlas (TCGA), recognizing it as the most extensive and systematically curated resource currently available for pan-cancer survival analysis.

To assess whether protein-level data might yield similar conclusions, we supplemented our transcriptomic analysis with a review of the limited immunohistochemical (IHC) studies available that have evaluated the association between Snail protein expression and patient survival.

Immunohistochemical analysis by Wang et al. corroborated our mRNA-based observations. In a cohort of 78 NSCLC patients with stage N0 who underwent surgical resection at Shandong Provincial Hospital, reduced Snail protein expression was significantly associated with improved survival (*P* = 0.007) [112]. This finding is consistent with the survival advantage observed in our transcriptomic analysis for patients with low *SNAI1* mRNA expression (*P* = 0.023). 

Immunohistochemical analysis performed on colon tumor tissues and adjacent non-pathological mucosa showed that overall survival in the group of patients with low levels of Snail expression was significantly higher than in patients with moderate or high levels of Snail immunoreactivity (*P* < 0.001) [113]. This finding is consistent with the survival data obtained from our analysis of *SNAI1* mRNA expression in the TCGA cohort (https://portal.gdc.cancer.gov, Fig. 2) (*P* = 0.005).

He et al. investigated the prognostic significance of Snail protein expression in a cohort of 103 gastric cancer patients. Survival analysis revealed that patients with high Snail expression had significantly poorer overall survival compared to those with low Snail expression (*P* < 0.001) [114]. This finding is consistent with our analysis of *SNAI1* mRNA expression in the STAD cohort from the TCGA database (*P* = 0.009) (https://portal.gdc.cancer.gov, Fig. 2), which also demonstrated a significant association between elevated *SNAI1* mRNA expression and reduced patient survival.

In pharyngeal squamous cell carcinoma, patients with low Snail protein expression exhibited significantly better overall survival compared to Snail-positive patients (*P* = 0.014) [115]. Given the established epidemiological link between pharyngeal and esophageal squamous cell carcinoma [116], similar survival outcomes might be expected in both malignancies. Consistent with this notion, our analysis of *SNAI1* mRNA expression in the ESCA cohort revealed a significant association between elevated transcript levels and reduced patient survival (*P* = 0.011), paralleling the prognostic value of Snail protein expression reported in pharyngeal cancer.

In ovarian cancer, immunohistochemical analysis revealed that patients with positive Snail protein expression had significantly shorter overall survival (*P* = 0.047) [117]. In contrast, our analysis of *SNAI1* mRNA expression using TCGA data failed to demonstrate a significant association between transcript levels and patient outcomes (*P* = 0.24) (https://portal.gdc.cancer.gov, Fig. 2).

A striking discordance between protein and mRNA data was observed in hilar cholangiocarcinoma. While immunohistochemical analysis of 47 patients demonstrated a highly significant association between high Snail expression and poor survival (*P* < 0.0001) [118], *SNAI1* mRNA levels from the GDC portal (https://portal.gdc.cancer.gov) showed no significant correlation with patient outcomes (Fig. 2).

Hepatocellular carcinoma (HCC) presented another notable discordance between protein and mRNA data. Immunohistochemical analysis of HCC tissues revealed that high Snail protein expression was significantly associated with poor patient survival (*P* = 0.034). In contrast, elevated *SNAI1* mRNA expression (data from https://portal.gdc.cancer.gov) is not associated with worse survival in LIHC patients (*P* = 0.59; Fig. 2).

In renal cell carcinoma (RCC), immunohistochemical analysis revealed that the level of Snail was not associated with patient survival (*P* = 0.493) [119]. In contrast, *SNAI1* mRNA expression data from the GDC Data Portal (https://portal.gdc.cancer.gov) demonstrated significant correlations with prognosis in both KIRP and KIRC cohorts (*P* = 0.04 and *P* = 0.02, respectively). It should be noted, however, that direct comparisons between protein and mRNA data obtained through different methodologies and from distinct patient cohorts require cautious interpretation.

In summary, the concordance between Snail mRNA and protein expression in predicting patient survival varied across cancer types. Strong agreement was observed in NSCLC, COAD, STAD, and ESCA. In contrast, discrepancies between transcript- and protein-level prognostic value were evident in OV, HCC, and CHOL. However, it should be noted that the comparisons for BLCA and RCC may be less reliable due to methodological or cohort limitations. Future studies incorporating systematic immunohistochemical evaluation of Snail expression in large, clinically annotated cohorts are warranted to clarify its true prognostic value.

### Snail targets confirmed by ChIP assay

Since Snail is a transcription factor, it modulates gene expression by binding to specific E-box motifs in the promoter regions of its target genes.

Snail affects the expression of numerous genes, which may be its transcriptional targets. These genes have been described elsewhere in the literature. In this review, we selected genes for which Snail binding to their regulatory regions had been demonstrated using ChIP assay and verified by other techniques, such as the luciferase assay or the electrophoretic mobility shift assay (EMSA) (Table 1). With this approach, we can be confident that these genes are indeed the direct transcriptional targets of Snail. Notably, these genes link the effect of Snail with EMT, metastasis, CSC, and resistance to chemotherapeutics.

**Table 1:** The list of Snail targets confirmed by the ChIP assay

Gene	Effect of Snail Binding	Key Function	Reference
*E-cadherin (CDH1)*	Repression	Calcium-dependent intercellular adhesion; prevents migration/invasion.	[28]
*ZEB1*	Activation (with EGR1/SP1)	Key regulator of EMT, tumor development, and metastasis.	[120]
*PTEN*	Repression	Tumor suppressor inhibiting the PI3K pathway.	[57]
*Occludin (OCLN)*	Repression	Critical integral membrane protein for tight junction integrity.	[121]
*MMP9*	Activation (with EGR1/SP1)	Matrix metalloproteinase that degrades ECM, enhancing invasion.	[120]
*Fibronectin 1 (FN1)*	Activation (with p65 NF-κB)	Major ECM glycoprotein involved in adhesion and migration.	[122]
*CXCL1 & CXCL2*	Activation	Chemokines attracting MDSCs to tumors, suppressing immunity.	[60]
*COL1A1*	Activation (with EGR1/SP1)	Major structural collagen of ECM; associated with metastasis.	[123]
*Slug (SNAI2)*	Repression	SNAIL family TF linked to cell survival and EMT.	[124]
*PFKP*	Repression	Rate-limiting glycolytic enzyme; repression shifts metabolism.	[125]
*PEBP1 (RKIP)*	Repression	Tumor suppressor inhibiting Raf-MEK-ERK and NF-κB pathways.	[126]
*LLGL2*	Repression	Regulates cell polarity; implicated in metastasis.	[127]
*CYLD*	Repression	Deubiquitinase and tumor suppressor regulating multiple pathways.	[128]
*Crumbs3 (Crb3)*	Repression	Transmembrane protein promoting apicobasal polarity.	[129]
*PTGS2 (COX-2)*	Activation (with EGR1/SP1)	Key enzyme in prostaglandin biosynthesis (inflammation).	[130]
*Maspin (SerpinB5)*	Repression	Serine protease inhibitor reducing invasion and migration.	[131]
*LEF1*	Activation (with EGR1/SP1)	Transcription factor in WNT signaling; crucial for EMT.	[132]
*FBP1*	Repression	Suppresses glycolysis; tumor suppressor via multiple mechanisms.	[133]
*EIF4EBP1*	Repression	Translational repressor; associated with metastasis.	[134]
*EPHB3*	Repression	Receptor tyrosine kinase; context-dependent tumor suppressor.	[135]
*CXADR*	Repression	Part of tight junction complex; role in cancer is controversial.	[136]
*VDR*	Repression	Vitamin D receptor; associated with better survival.	[137]
*BID*	Repression	Pro-apoptotic protein (Bcl-2 family); generally a tumor suppressor.	[138]
*DFFB*	Repression	Apoptotic DNase; enhances mutagenesis and drug resistance.	[138]
*EGR1*	Repression	Transcription factor; role in cancer is context-dependent.	[139]
*ESR1*	Repression	Encodes estrogen receptor-alpha; has an important role in breast cancer development.	[140]
*ESRP1*	Repression	Splicing regulator that maintains epithelial phenotype.	[141]
*KRT17*	Repression	Keratin protein; high expression linked to poor prognosis.	[142]
*KRT18*	Repression	Keratin protein; high expression linked to poor prognosis.	[142]
*OTUD7A*	Repression	Deubiquitinase; predicted tumor suppressor.	[143]
*PRRX1*	Repression	Homeobox transcription factor; considered an oncogene.	[144]
*RAB25*	Repression	Small GTPase; induces EMT through specific signaling axis.	[142]
*SLC27A2*	Repression	Fatty acid transporter; acts as a tumor suppressor.	[142]
*THBD*	Repression	Thrombomodulin; involved in coagulation and inflammation.	[145]

#### Negative feedback regulatory loop between SNAIL and TP53

A number of studies have firmly established that EMT and the major tumor suppressor p53 are inversely correlated [146, 147]. Importantly, K-Ras mutant-expressing epithelial cancer cells (lung cancer, pancreatic and colorectal cancers) also contain the Snail protein [148–150]. It is plausible that overexpressed or stabilized Snail in turn may activate the expression of Zeb1 in these tumor cells. Unlike Zeb1, which represses the *TP53* gene expression [3], Snail can suppress the tumor-suppressive function of p53 by augmenting the level of Mdm2 expression [21]. This promotes p53 ubiquitinylation and its subsequent degradation by the proteasomes [151], thereby inhibiting its epithelial-protective activity. Intriguingly, wild-type p53 was also shown to trigger Snail degradation via the MDM2 pathway, highlighting a reciprocal regulatory loop that is frequently disrupted in cancer [16].

Furthermore, Kajita et al. demonstrated using ChIP assay that Snail binds to an E-box (CANNTG) in the *TP53* promoter and represses its expression in the MCF7 cell line [138].

### The relationship between *SNAI1* and expression levels of its direct transcriptional targets in different types of malignancies

**Fig. 3:**
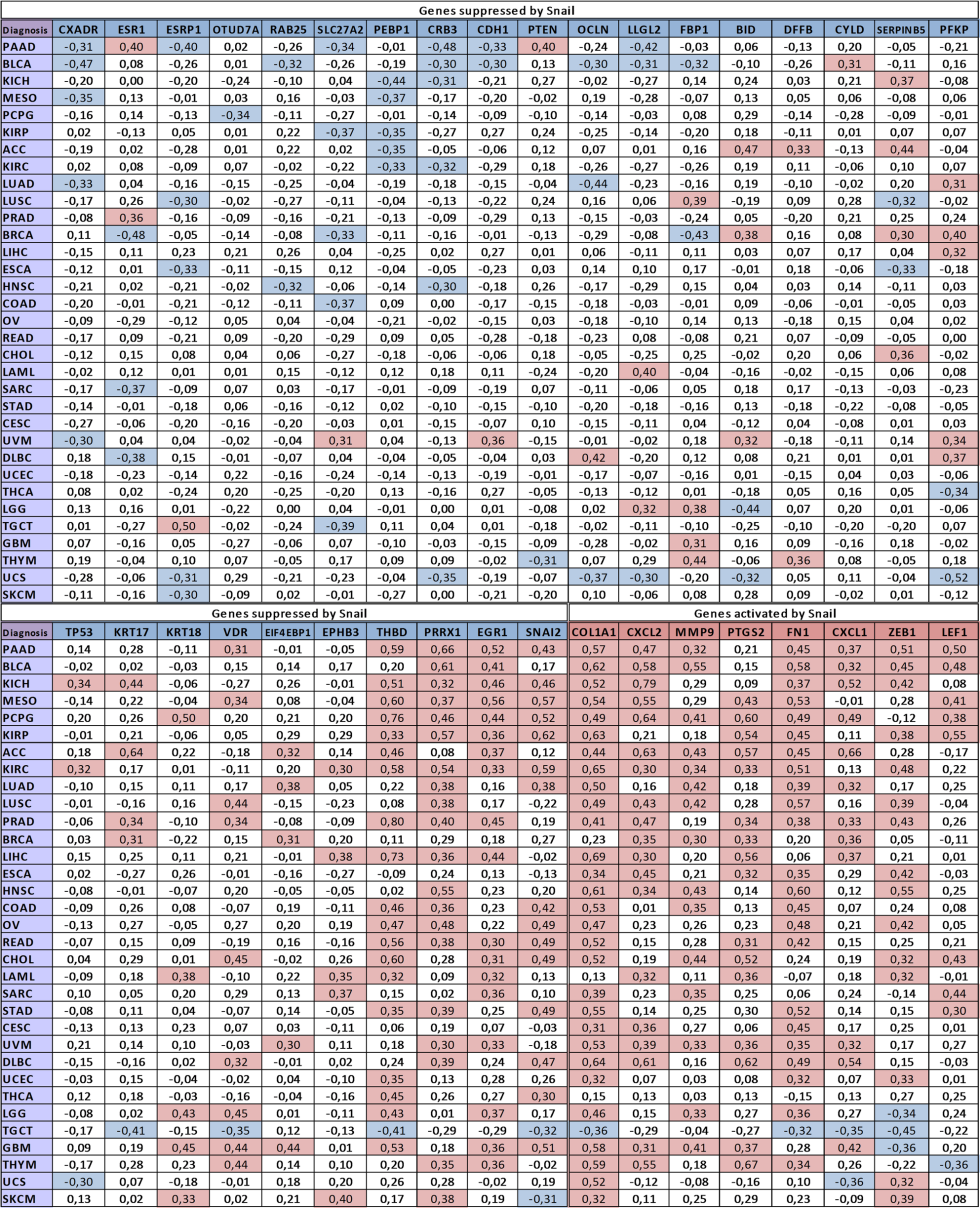
The Pearson correlation coefficient between *SNAI1* expression and its target genes is shown. The corresponding p-values are provided in Supplementary Table 1. Genes repressed (blue) and activated (red) by *SNAI1* are indicated. The correlation coefficients for each malignancy type are shown below. Blue denotes a negative correlation, and red denotes a positive correlation, between *SNAI1* expression and its target genes. Patient gene expression data were obtained from https://portal.gdc.cancer.gov/

Using data from https://portal.gdc.cancer.gov, we found a correlation between the expression of *SNAI1* and its target genes in various malignancies. The Pearson correlation coefficients are shown in Fig. 3, and the P values for each correlation coefficient are reported in Supplementary Fig. 1.

For Snail-activated targets, a high correlation (r *≥* 0.3) is observed in most cancer types (Fig. 3). In contrast, the pattern for repressed genes (Fig. 3) is more complex: among 33 malignancies, only a few exhibit a strong negative correlation between *SNAI1* expression and its putative transcriptional targets. For instance, a negative correlation between *SNAI1* and *CDH1* (encoding E-cadherin) is observed only in pancreatic adenocarcinoma (PAAD) and bladder urothelial carcinoma (BLCA), with no such relationship detected in other diseases. Furthermore, for several genes considered to be negatively regulated by Snail (*EGR1*, *PRRX1*, *THBD*, *EIF4EBP1*, *VDR*, *CYLD*, *PFKP*, *SNAI2*), a positive correlation with *SNAI1* expression is observed (Fig. 3). This phenomenon is particularly evident for *SNAI2*: although Snail acts as a negative regulator of *SNAI2*, its expression increases upon *SNAI1* induction. Taken together, these findings indicate that Snail tightly regulates its positively controlled targets, whereas in the context of negative regulation, additional factors may modulate or counteract the direct inhibitory effect of Snail.

Thus, a difference was observed between the groups of genes negatively and positively regulated by Snail. In most cases, Snail represses its target genes by binding to E-box motifs (CANNTG) within their regulatory regions. In contrast, Snail activates transcription by interacting with the non-canonical TCACA sequence, in cooperation with two additional transcription factors, EGR1 and SP1.

In this study, among the Snail target genes analyzed, six mesenchymal genes - *FN*, *LEF*, *MMP9*, *ZEB1*, *COX2*, and *COL1A1-* were shown to be positively regulated by Snail through its interaction with Early Growth Response Factor 1 (EGR1) and Specificity Protein 1 (SP1) [130]. EGR1 is a transcription factor that is involved in the repair of tissue damage and fibrosis and is associated with cancer initiation and progression [152]. The transcription factor SP1 is required for the expression of a large number of housekeeping genes, many of which play important roles in the initiation and development of cancer [153]. The authors noted that the Snail binding motif (TCACA) is located in the promoter region upstream of the EGR/SP1 overlapping binding sites, which is true for the above-mentioned six genes. Similar situation was observed for several other genes: (*VIMENTIN* (*VIM*), *VITRONECTIN* (*VTN*), *Actin Alpha 2*, Smooth Muscle (*α-SMA*), N-cadherin (*CDH2*), *TWIST1*), which also contribute to the acquisition of a mesenchymal phenotype by the cell [130]. Moreover, the authors proposed a model for Snail-dependent up-regulation of mesenchymal genes through cooperation with EGR1 and SP1 [130]. This may explain the absence of the CAGGTG sequence in the promoter region of a numerous genes regulated by Snail.

### Predictive Snail binding motifs in the regulatory regions of its target genes

**Fig. 4:**
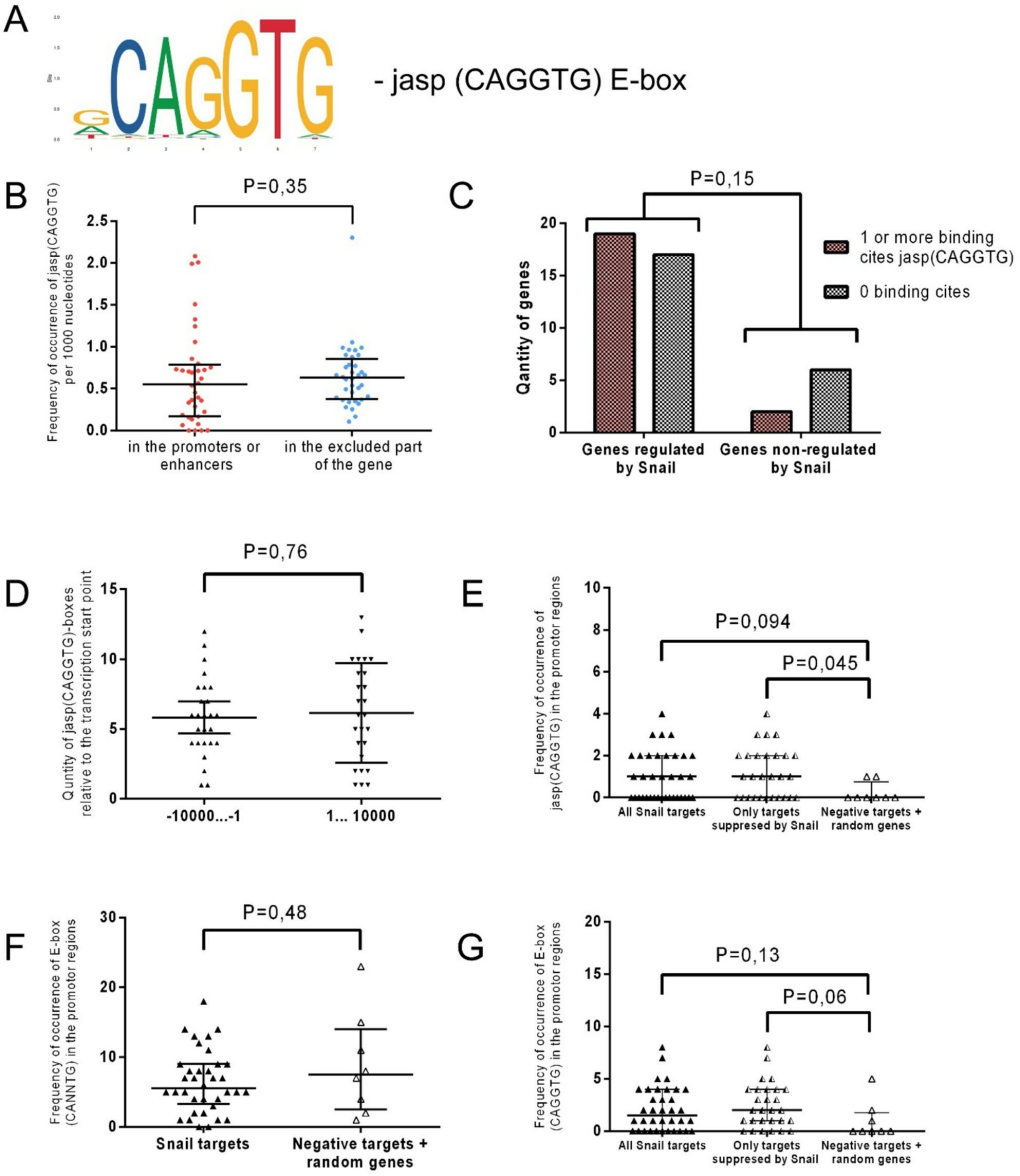
Statistical analysis of Snail binding site distribution within its target genes. Data were obtained from https://rest.ensembl.org/. **A**. The frequency matrix of the Snail transcription factor binding motif — Jasp(CAGGTG)-Ebox — was obtained from the JASPAR 2024 database (https://jaspar.elixir.no/). **B**. Comparison of Jasp(CAGGTG)-box frequency in promoter regions and enhancers versus other parts of the gene. Median and 25th/75th percentiles are shown. N = 36. A Mann-Whitney U test was used. **C**. Distribution of genes containing one or more JASPAR CAGGTG boxes versus genes containing none, in both Snail-regulated and non-Snail-regulated groups. A chi-square test was used. **D**. Comparison of the number of Jasp(CAGGTG)-boxes located upstream and downstream of the transcription start site (TSS) per gene. Mean and standard deviation are shown. *N* = 26. A t-test was used. **E**. Comparison of promoter regions enriched for Jasp(CAGGTG)-boxes across three gene groups: all Snail-regulated targets, Snail-repressed targets, and genes not regulated by Snail. Nmin = 8. Median and 25th/75th percentiles are shown. A Mann-Whitney U test was used. **F**. Comparison of promoter region enrichment for (CANNTG)-boxes between two gene groups: all Snail-regulated targets and genes not regulated by Snail. Nmin = 8. Median and 25th/75th percentiles are shown. A Mann-Whitney test was used. **G**. Comparison of promoter region enrichment for (CAGGTG)-boxes across three gene groups. Nmin = 8. Median and 25th/75th percentiles are shown. A Mann-Whitney test was used

During this study, we observed that Snail binding sites defined by the Jasp(CAGGTG) motif (https://rest.ensembl.org/) may be more relevant for predicting potential Snail targets than the classical E-box (CANNTG) sequence. To test this hypothesis, we performed a statistical analysis of the distribution of these sequences within genes. Then, we compared the frequency with which they occurred in the promoters of Snail-regulated versus non-regulated genes. Specifically, we analyzed the sequence of Snail target genes, as well as the upstream non-coding region extending to -10,000 bp, for the presence of Jasp(CAGGTG) sites (Fig. 4A).

We found no preferential location of Jasp(CAGGTG) sites in the promoter and enhancer regions; they were randomly distributed throughout the gene region (Fig. 4B). Furthermore, no difference was observed in the number of Jasp(CAGGTG) relative to the transcriptional start site (TSS) within 20,000 bp (Fig. 4D). No significant difference was observed when comparing the number of genes containing the Jasp(CAGGTG) motif in the promoter region between Snail-regulated genes and control genes (non-regulated by Snail) (Fig. 4C).

Since Snail can regulate its target genes not only through the classical CAGGTG E-box (primarily associated with gene repression) but also via the TCACA sequence (primarily associated with gene activation), we further analyzed the dataset after excluding Snail-activated genes. Under this condition, the groups (Snail-regulated vs. control genes) differed significantly in the number of Jasp(CAGGTG) boxes present in the promoter region (*P* = 0.045) (Fig. 4E). Since Snail binds not only to the canonical CAGGTG E-box but also to other variants, such as CANNTG, we compared the number of CANNTG E-boxes in the target genes and the negative control (Fig. 4F). No significant difference was found when comparing validated Snail targets and controls (*P* = 0.48).

The difference between the groups became more pronounced when the E-box sequence CANNTG compressed to CAGGTG, though it remained non-significant (*P* = 0.13) (Fig. 4G). When the target gene sample was further restricted by excluding Snail‑activated genes, the difference became more significant.

Therefore, predicting Snail’s potential targets based solely on the presence of CAGGTG or CANNTG E-box motifs is not reliable. Thus, it is preferable to analyze the number of JASPAR CAGGTG boxes in the promoter region. However, this approach will not capture cases where Snail interacts with CANNTG E-boxes.

For example, the *SNAI2*,* PEBP1*,* THBD*,* RAB25*,* PRRX1*,* EIF4EBP* and *SERPINB5* promoters lack Jasp(CAGGTG)-boxes but contain a CANNTG E-box through which Snail interacts. Furthermore, the *OTUD7A*,* DFFB*, and *BID* promoters lack all variants of E-boxes, including Jasp (CAGGTG), CAGGTG, and CANNTG. Consequently, identifying potential Snail targets based solely on the presence of Jasp(CAGGTG)-boxes has inherent limitations. Conversely, searching for targets using the CANNTG motif is complicated by its near-ubiquitous presence across virtually all human genes. Narrowing the search to the more specific CAGGTG sequence improves efficiency but results in the loss of some genuine targets [154]. Using the Jasp(CAGGTG) algorithm to search further narrows the list of potential targets, which makes the search more reliable; however, this also results in the loss of some targets. Additionally, it should be noted that searching for the aforementioned sequences will not reveal Snail’s positively regulated targets, which constitute approximately 15% of all genes directly regulated by this factor. Therefore, a more advanced method for searching for potential Snail targets is needed, possibly incorporating the above-discussed search algorithms.

**Fig. 5:**
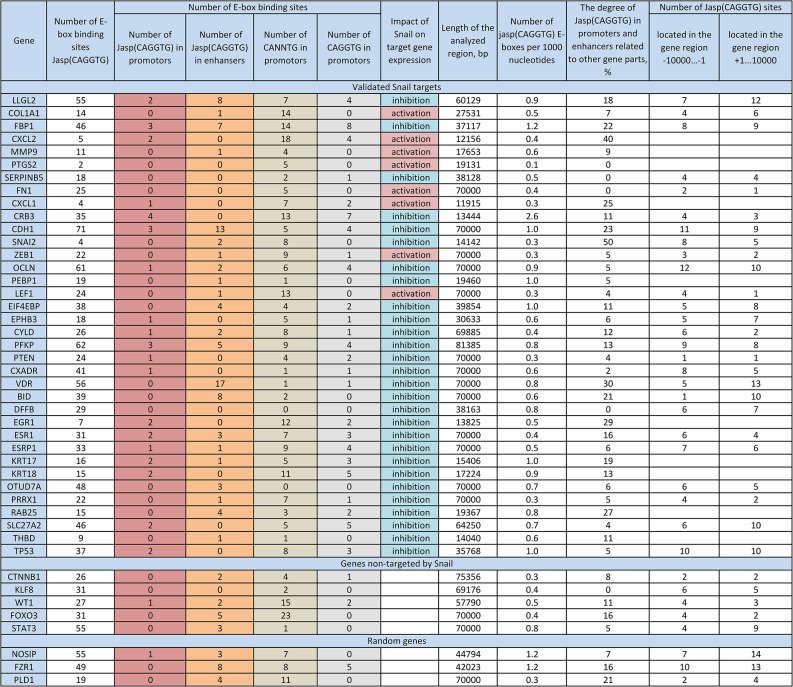
Frequency of CAGGTG, CANNTG, and Jasp(CAGGTG) motifs within E-box regions of genes as potential Snail binding sites. Genes are categorized into three groups: validated Snail targets, genes non-targeted by Snail, and a random set of human genome genes serving as a control. For statistical analysis, the latter two groups were combined into a single category, “Negative targets and random genes.”  The table presents the number of E-box sites (CAGGTG, CANNTG, and Jasp(CAGGTG)) in the promoters and enhancers of the listed genes. The column “Effect of Snail on the target” indicates the type of regulation (activation or repression) exerted by Snail. “Number of Jasp(CAGGTG) E-boxes per 1000 nucleotides” is calculated by dividing the number of Jasp(CAGGTG) binding sites by the length of the analyzed gene region and multiplying by 1000, providing the average frequency of this motif within the gene. “Distribution of Jasp(CAGGTG) across promoters and enhancers (%)” represents the percentage of all Jasp(CAGGTG) sites found within promoter and enhancer regions, reflecting the relative importance of these regulatory elements for Jasp(CAGGTG) binding

**Fig. 6:**
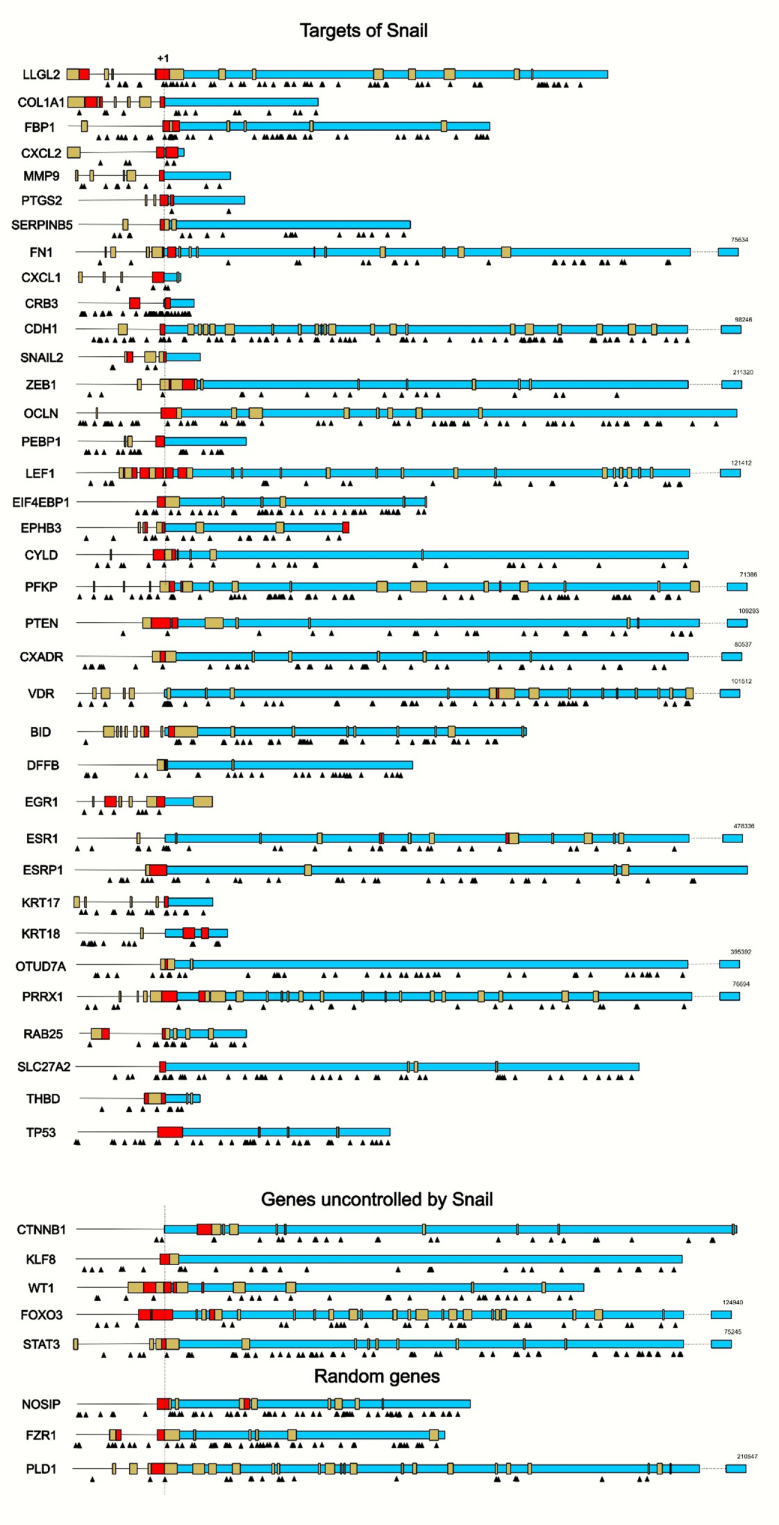
Analysis of Snail binding site distribution in its target genes. A schematic comparison is presented for three groups of genes: those directly regulated by Snail (Targets of Snail), non-regulated genes (Genes uncontrolled by Snail), and randomly selected genes (Random genes). The gene body is shown in blue, promoters in red, and enhancers in yellow. “+1” indicates the transcriptional start site (TSS), and the triangle represents the Jasp(CAGGTG) motif. Data were obtained from https://rest.ensembl.org/

More detailed information on the distribution of E-boxes within Snail target genes is presented in Figs. 5 and 6.

It is important to note that most genes activated by Snail are regulated via its binding the TCACA sequence. This process may also be facilitated by the transcription factors EGR1 and SP1, which bind to the E/S sites located near the TCACA sequence. Therefore, the most effective way to search for potential Snail transcriptional targets is to look for Jasp(CAGGTG)-boxes and TCACA-E/S sites in the promoter region. However, genes that interact with Snail via CANNTG E-boxes should be searched for differently. Any final conclusions about direct Snail regulation should be validated using ChIP assay coupled with an additional method, such as EMSA.

It is also worth noting that Snail can regulate its target genes via the YAP/TAZ/TEAD complex, which binds the TEAD sequence, thereby enhancing the expression of CTGF, AXL, Ankrd1, and Dkk1, as well as through the GC-rich element of the FN1 gene via binding to the NF-κB/PARP1 complex [7].

**Fig. 7:**
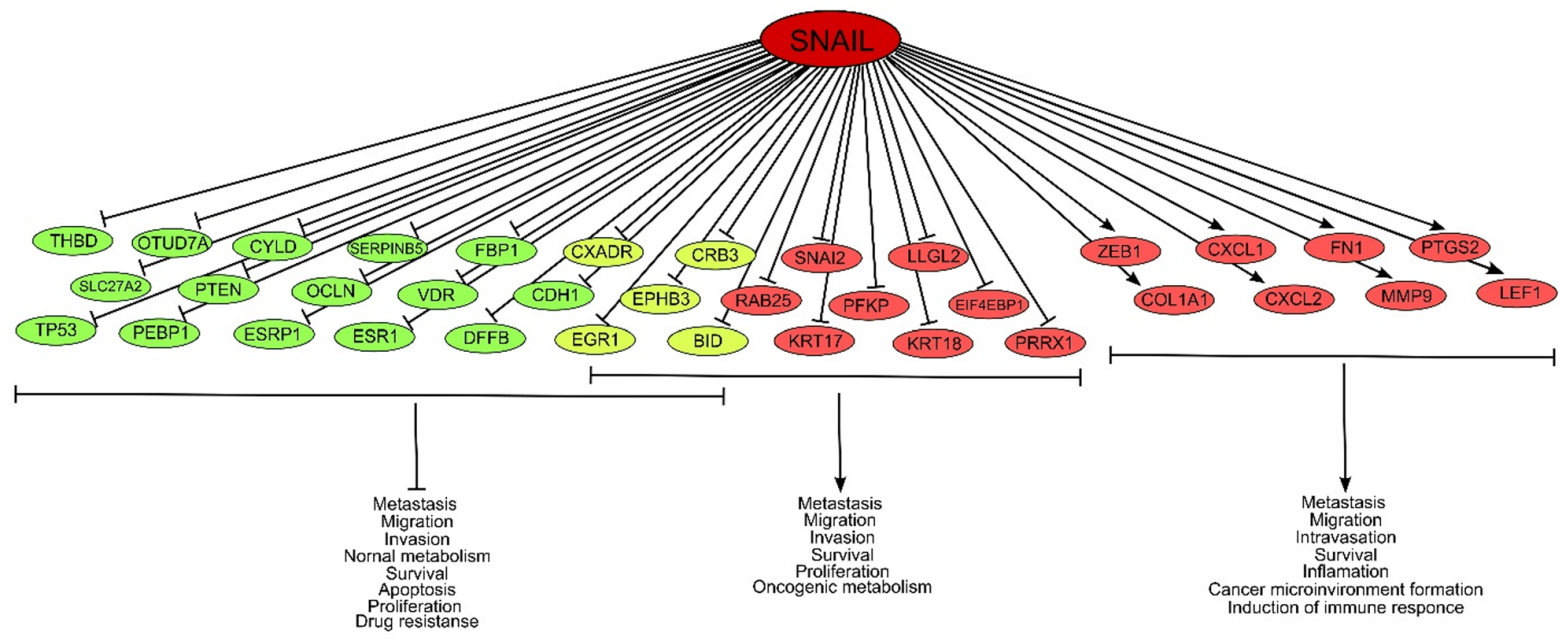
Scheme of Snail‑mediated regulation of cancer-associated genes

Snail target genes can generally be divided into three groups (Fig. 7). The first group includes tumor suppressors, such as *CYLD*,* SERPINB5*,* FBP1*,* PEBP1*,* PTEN*,* OCLN*,* THBD*,* OTUD7A*,* SLC27A2*,* VDR*,* TP53*,* ESRP1*,* ESR1*,* DFFB* and *CDH1*, which are negatively regulated by Snail. The second group includes oncogenes, such as *ZEB1*,* CXCL1*,* FN1*,* PTGS2*,* COL1A1*,* CXCL2*,* MMP9*, and *LEF1*, which are positively regulated by Snail. The third group includes oncogenes, such as *SNAI2*,* LLGL2*,* RAB25*,* EIF4EBP1*,* KRT17*,* KRT18*,* PPRX1* and *PFKP*, which are suppressed by Snail. *CXADR*,* EPHB3*,* EGR1*,* BID* and *CRB3* act as both oncosuppressors and tumor promoters, so they can be attributed to groups one and three.

Thus, Snail inhibits tumor suppressors and activates oncogenes, promoting cancer development. However, due to the presence of tumor suppressors, Snail can also suppress the development of cancer. This group of effectors may allow Snail to positively affect tumors in certain types of tissue.

#### Snail inhibitors

Given the accumulated knowledge regarding the role of Snail in metastasis, cancer stemness and resistance to therapy, targeting Snail in patients would be a promising strategy.

There are four known substances (Co^III^-Ebox, GN25, GN29, and CYD19) that are able directly bind Snail (Table 2). However, they have not been studied in clinical trials, and the preclinical data regarding them are limited.

Many other compounds affect Snail indirectly by different mechanisms. However, in the present review, we want to focus on several compounds, suppressing the activity of principal Snail’s cofactors mediating its transcriptional modulation (inhibitors of LSD1, HDACs, EZH2) (Table 2). Two of them (parnate and entinostat) have been extensively studied in clinical trials.

#### Compounds that affect Snail directly 

Inhibiting transcription factors like Snail is a notoriously challenging task due to the lack of traditional “druggable” pockets for small molecules in these proteins. However, four compounds have been found to directly bind and inhibit Snail.

**Co**^III^**-Ebox** is a cobalt(III) complex attached to an E-box oligonucleotide (CAGGTG) that confers specificity towards Snail TFs. This substance inhibits Snail binding to target DNA, with approx. 10-fold specificity over scrambled oligonucleotide control. Its efficacy has been demonstrated in vitro only in SKBR3 and MCF7 breast cancer cell lines [155]. Although this substance could potentially be used as an antimetastatic therapy to limit the effects of Snail by acting as its inhibitor by directly binding to it, up to date, there are no in vivo data on its efficiency and safety. Given our aforementioned data on E-box distribution across Snail-targeted genes, its targeting may not be efficient to suppress the plethora of genes which are specifically affected by Snail. However, E-box targeting may also suppress genes which are modulated by Zeb1 [3].

For potential therapeutic application, the main challenge seems to be the development of an effective nuclear delivery approach. In addition, to avoid the general off-target toxicity, this substance should be “unpacked” in tumor but not in other tissues. Furthermore, cobalt compounds that release cobalt ions in vivo are reasonably anticipated to be human carcinogens [156].

GN25 and GN29 are small chemical inhibitors that block the binding of the Snail and p53 proteins. Snail can suppress the tumor suppressor p53 in certain cancers. By inhibiting this interaction, GN25 and GN29 can restore the function of p53, resulting in anti-cancer effects such as reduced cell viability, reversal of the EMT phenotype and induced apoptosis [157]. These compounds are particularly effective against cancers with K-Ras mutations and Snail overexpression, such as pancreatic and lung cancers, because they specifically target the Snail-p53 interaction in these cells. Unlike GN29, GN25 is water-soluble and more effective. It reverses EMT, reactivates p53 and broadly reprograms oncogenic transcription networks (inhibits Myc, TBX2; activates tumor suppressor TFs) [158]. In vivo experiments in mice showed that it suppresses tumor growth effectively, leading to regression [157].

CYD19 is a potent dual-target inhibitor of both Snail and HDAC. With an IC₅₀ of 0.405 µM against HDAC1 and 0.18 µM against Snail, CYD19 exhibits strong inhibitory activity. To cause cell death, CYD19 decreases Snail protein levels and increases histone H4 acetylation [159].

Binding of CYD19 to Snail disrupts its interaction with CREB-binding protein (CBP)/p300. This inhibits CBP/p300-mediated acetylation of Snail’s targets. Furthermore, CYD19 promotes Snail’s ubiquitin-proteasome degradation [160]. CYD19 has been shown to reduce the migratory potential of MMTV-PyMT, HCT116, and SUM159 cell lines. CYD19 has also been shown to reduce Snail-induced tumor growth, pulmonary metastasis, and CSC expansion in MMTV-PyMT transgenic mice [160]. Regarding Co^III^-Ebox, GN25, and GN29, only preclinical data are available for CYD19.

**Table 2:** Several compounds that affect Snail directly, or inhibit its main cofactors. A description is provided in the text

Compound	Molecular mechanism	Reference
Co^III^-Ebox	Directly binds Snail and prevents its interaction with DNA	[155]
GN25 and GN29	Inhibit Snail-p53 interaction, increase the level of p53 and uncouple it from Snail	[157]
Parnate	Blocks Snail/Slug-LSD1 interaction by inhibition of LSD1	[161]
CYD19	Binds Snail and disrupts its interaction with CBP/p300, leading to Snail degradation	[160]
Entinostat	Inhibits the activity of HDAC1 and directly inhibits Snail-stimulated efflux activity of P-glycoprotein	[162]

#### Compounds affecting main Snail’s cofactors (LSD1, HDAC1, EZH2)

**Tranylcypromine (parnate).** The invasive process of EMT in cancer cells is critically dependent on the interaction between Snail/Slug transcription factors and lysine-specific demethylase 1A (LSD1, also known as KDM1A), a demethylase that removes methyl groups from H3K4me1/2 and H3K9me1/2 histones [161] and hence regulates gene expression [163]. Disrupting the function of the Snail/Slug-LSD1 complex using the LSD1 inhibitor tranylcypromine (parnate) effectively suppresses cancer cell motility and invasion by reversing EMT without affecting cell proliferation. Therefore, the Snail/Slug-LSD1 complex is a promising therapeutic target for the specific inhibition of cancer cell invasiveness. However, it should be noted that parnate is approved by the U.S. Food and Drug Administration for the treatment of severe depression, and its use can cause side effects including increase in blood pressure, increased levels of serotonin, dopamine, epinephrine, and norepinephrine [161, 164].

Tranylcypromine (Parnate) is rapidly absorbed, reaching peak plasma concentrations (Cₘₐₓ ~50–150 ng/mL) within 1–2 h (Tₘₐₓ) after a single 20 mg dose [165, 166]. Despite a short plasma half-life of 1.5–3 h, its clinical effect is prolonged because it irreversibly inhibits monoamine oxidase, an action that persists for days until new enzyme is synthesized [167]. The drug exhibits non-linear kinetics and significant inter-individual variability in exposure, which necessitates careful dose titration to manage associated hypertensive crisis [166].

However, there have been no clinical trials on the use of tranylcypromine in cancer therapy. In contrast, a set of tranylcypromine-based derivatives has been developed, some of which (iadademstat (ORY-1001), Bomedemstat (IMG-7289), INCB059872, CC-90011) have been studied in clinical trials (phases I–II), primarily against acute myeloid leukemia (AML) [168–171]. However, their impact on Snail remains to be elucidated.

**Entinostat** (also known as SNDX-275 and MS-275) is an oral, a benzamide class I-selective histone deacetylase (HDAC) inhibitor that is being studied in clinical trials to treat a variety of malignancies. It has an IC50 of 0.51 µM for HDAC1 and 1.7 µM for HDAC3 [172].

Snail recruits HDACs to the promoters of its targets [159, 173–175], forming a repressor complex that silences their expression. HDAC activity removes acetyl groups from histones, creating a condensed, transcriptionally inactive chromatin structure that prevents transcription. This HDAC-dependent epigenetic repression is one of the fundamental mechanisms by which Snail initiates EMT, enhancing tumor cell invasion and metastasis. Entinostat reverses EMT in breast cancer by downregulating Snail [162]. Additionally, it reverses Snail-induced EMT in lung cancer cells, thereby suppressing their migratory capacity. Furthermore, entinostat directly inhibits Snail-stimulated efflux activity of P-glycoprotein (P-gp), a key mediator of multidrug resistance [176]. Together, these findings suggest that entinostat has dual therapeutic potential, as it simultaneously targets epigenetic regulation and overcomes chemotherapy resistance.

Entinostat is not yet approved by FDA but has been extensively studied in Phase 2 and 3 trials [177, 178], most notably in combination with hormonal therapy for advanced estrogen receptor-positive (ER+) breast cancer [179]. The combination of exemestane (aromatase inhibitor) + entinostat showed a significant improvement in progression-free survival and overall survival in postmenopausal women with ER+/HER2- advanced breast cancer who had progressed on prior non-steroidal aromatase inhibitor therapy, as demonstrated in the ENCORE 301 trial [180].

Entinostat has a pharmacokinetic profile well-suited for once-weekly dosing. After oral administration, the drug is rapidly absorbed, reaching its maximum plasma concentration (Cmax) within 1 to 3 h (Tmax). The mean Cmax for the clinically used 5 mg weekly dose is approximately in the range of 100 to 200 nM. It has a very long terminal half-life, which ranges from approximately 40 to over 150 h. This extended half-life is the rationale behind the weekly dosing schedule, as it ensures sustained target coverage throughout the dosing interval. Pharmacokinetic data are derived from foundational Phase 1 studies [181, 182].

**EZH2 inhibitors**. Snail recruits the PRC2 complex, specifically EZH2, to target gene promoters like *CDH1*, where EZH2 catalyzes H3K27me3 deposition, leading to stable epigenetic repression of epithelial genes [183]. The lncRNA HOTAIR acts as a specific adaptor, enabling the functional cooperation between Snail and EZH2 by forming a tripartite complex that recruits the chromatin-modifying PRC2 machinery to Snail target genes, thereby executing stable epigenetic repression essential for EMT and hepatocyte transdifferentiation [184].

Since EZH2 is an important co-factor of Snail, inhibiting it should significantly affect Snail-mediated transcriptional reprogramming. Tazemetostat (Tazverik^®^) is an oral, selective inhibitor of EZH2, which is the catalytic subunit of Polycomb Repressive Complex 2 (PRC2). It blocks the enzyme’s ability to deposit the repressive H3K27me3 histone mark. It is an FDA-approved drug for treating epithelioid sarcoma and follicular lymphoma. Despite its broad use in various clinical trials, the impact of tazemetostat on Snail-induced changes in transcription remains unknown.

Although direct, high-affinity small-molecule inhibitors of Snail remain elusive for clinical application, the most clinically advanced strategies instead employ HDAC and LSD1 inhibitors. These agents effectively neutralize Snail function by inhibiting the epigenetic repressor complexes with which Snail interacts.

## Conclusions and future perspectives

- For most malignancies, high Snail levels are a negative prognostic marker. However, this correlation is not universal; it is true for certain tissue types (e.g., digestive tract, kidney, lung) but absent in others (e.g., breast, liver, pancreatic cancers).
- Direct validation by Chromatin Immunoprecipitation (ChIP) assay is crucial for confirming functional interactions between Snail and its transcriptional targets.
- While Snail is known to bind E-box sequences, it shows a strong preference for the CAGGTG motif over the generic CANNTG.
- The Jasp(CAGGTG) E-box sequence modulates gene expression only when located in the promoter region of Snail target genes.
- An alternative binding mechanism exists where Snail interacts with a TCACA motif via cooperation with adjacent EGR1/SP1 sites.
- A refined model for predicting Snail targets - prioritizing Jasp(CAGGTG) and TCACA sites is proposed to enable more effective drug discovery.
- According to our correlation analysis, Snail primarily exerts its oncogenic effects by directly activating a network of pro-metastatic and pro-survival oncogenes rather than by repressing epithelial genes.
- The most promising strategies for Snail suppression in clinical practice are the use of HDAC and LSD1 inhibitors, which effectively neutralize Snail’s function by disrupting its epigenetic repressor complexes, and the use of inhibitors of the TGFβ/ERK/NF-κB/Snail axis. Examples include parnate, entinostat, and disulfiram.

## Supplementary Information

Below is the link to the electronic supplementary material.

Supplementary Material 1: Supplementary Table 1. P-values for gene expression correlations. Link to Fig. 3.

Supplementary Material 2: Additional file 1. Methods.docx. The description of bioinformatics and statistical methods used.

## Data Availability

No datasets were generated or analyzed during the current study.

## Abbreviations

EMT: Epithelial-Mesenchymal Transition
CSC: Cancer Stem Cell
ECM: Extracellular Matrix
SNAI1: Snail Family Transcriptional Repressor 1 (Snail)
SNAI2: Snail Family Transcriptional Repressor 2 (Slug)
ZEB1/2: Zinc Finger E-Box Binding Homeobox 1/2
CDH1: Cadherin 1 (E-cadherin)
FN1: Fibronectin 1
MMP9: Matrix Metallopeptidase 9
LEF1: Lymphoid Enhancer Binding Factor 1
PTEN: Phosphatase and Tensin Homolog
CXCL1/2: C-X-C Motif Chemokine Ligand 1/2
COL1A1: Collagen Type I Alpha 1 Chain
FBP1: Fructose-Bisphosphatase 1
PEBP1/RKIP: Phosphatidylethanolamine Binding Protein 1/Raf Kinase Inhibitory Protein
CYLD: CYLD Lysine 63 Deubiquitinase
PTGS2/COX-2: Prostaglandin-Endoperoxide Synthase 2/Cyclooxygenase-2
SERPINB5: Serpin Family B Member 5 (Maspin)
EGR1: Early Growth Response 1
SP1: Specificity Protein 1
E-box: Enhancer box (CANNTG DNA sequence)
TSS: Transcription Start Site
TCGA: The Cancer Genome Atlas
GDC: Genomic Data Commons (NIH)
ChIP: Chromatin Immunoprecipitation
EMSA: Electrophoretic Mobility Shift Assay

## References

[1] Kielbik M, et al. Snail transcription factors as key regulators of chemoresistance, stemness and metastasis of ovarian cancer cells. Biochim et Biophys Acta (BBA)-Reviews Cancer. 2023;1878(6):p189003. 10.1016/j.bbcan.2023.189003 37863122

[2] Kaufhold S, Bonavida B. Central role of Snail1 in the regulation of EMT and resistance in cancer: a target for therapeutic intervention. J Experimental Clin Cancer Res. 2014;33(1):62. 10.1186/s13046-014-0062-0 PMC4237825 25084828

[3] Parfenyev SE, et al. Dualistic role of ZEB1 and ZEB2 in tumor progression. Biol Direct. 2025;20(1):32. 40114235 10.1186/s13062-025-00604-3 PMC11927373

[4] Parker HN, et al. Twist-Induced Epithelial-to-Mesenchymal Transition Confers Specific Metabolic and Mitochondrial Alterations. Cells. 2025;14(2):80. 39851508 10.3390/cells14020080 PMC11763985

[5] Saitoh M. Transcriptional regulation of EMT transcription factors in cancer. Seminars in cancer biology. Elsevier; 2023. 10.1016/j.semcancer.2023.10.001 37802266

[6] Epithelial-to-Mesenchyme Transition MSs. Pak1 Phosphorylation of Snail, a Master Regulator of. Cancer Res. 2005;65:3179–84. 15833848 10.1158/0008-5472.CAN-04-3480

[7] AG de Herreros. Dual role of Snail1 as transcriptional repressor and activator. Biochim et Biophys Acta (BBA)-Reviews Cancer. 2024;1879(1):189037. 10.1016/j.bbcan.2023.189037 38043804

[8] Motizuki M et al. The Snail signaling branch downstream of the TGF-β/Smad3 pathway mediates Rho activation and subsequent stress fiber formation. J Biol Chem. 2024;300(1). 10.1016/j.jbc.2023.105580 PMC10821601 38141763

[9] Dong J, et al. Activation of phosphatidylinositol 3-kinase/AKT/snail signaling pathway contributes to epithelial-mesenchymal transition-induced multi-drug resistance to sorafenib in hepatocellular carcinoma cells. PLoS ONE. 2017;12(9):e0185088. 28934275 10.1371/journal.pone.0185088 PMC5608310

[10] Cao Y-W, et al. Implications of the Notch1-Snail/Slug-epithelial to mesenchymal transition axis for lymph node metastasis in infiltrating ductal carcinoma. Kaohsiung J Med Sci. 2015;31(2):70–6. 25645984 10.1016/j.kjms.2014.11.008 PMC11916146

[11] Wu Y-d, Zhou B. TNF-α/NF-κB/Snail pathway in cancer cell migration and invasion. Br J Cancer. 2010;102(4):639–44. 20087353 10.1038/sj.bjc.6605530 PMC2837572

[12] Yook JI, et al. Wnt-dependent regulation of the E-cadherin repressor snail. J Biol Chem. 2005;280(12):11740–8. 15647282 10.1074/jbc.M413878200

[13] Carver EA, et al. The mouse snail gene encodes a key regulator of the epithelial-mesenchymal transition. Mol Cell Biol. 2001;21(23):8184–8. 11689706 10.1128/MCB.21.23.8184-8188.2001 PMC99982

[14] Jiang R, et al. The Slug gene is not essential for mesoderm or neural crest development in mice. Dev Biol. 1998;198(2):277–85. 9659933

[15] Kyuno D, et al. Role of tight junctions in the epithelial-to-mesenchymal transition of cancer cells. Biochim et Biophys Acta (BBA)-Biomembranes. 2021;1863(3):183503. 10.1016/j.bbamem.2020.183503 33189716

[16] Lim S-O, Kim H, Jung G. p53 inhibits tumor cell invasion via the degradation of snail protein in hepatocellular carcinoma. FEBS Lett. 2010;584(11):2231–6. 20385133 10.1016/j.febslet.2010.04.006

[17] Fedorova O, et al. The role of PTEN in epithelial–mesenchymal transition. Cancers. 2022;14(15):3786. 35954450 10.3390/cancers14153786 PMC9367281

[18] Wang S-P, et al. p53 controls cancer cell invasion by inducing the MDM2-mediated degradation of Slug. Nat Cell Biol. 2009;11(6):694–704. 19448627 10.1038/ncb1875

[19] Siemens H, et al. miR-34 and SNAIL form a double-negative feedback loop to regulate epithelial-mesenchymal transitions. Cell Cycle. 2011;10(24):4256–71. 22134354 10.4161/cc.10.24.18552

[20] Pan W et al. p53/MicroRNA-34 axis in cancer and beyond. Heliyon, 2023. 9(4). 10.1016/j.heliyon.2023.e15155 PMC10121403 37095919

[21] Barlev N, et al. The microRNA and p53 families join forces against cancer. Cell Death Differ. 2010;17(2):373–5. 20062068 10.1038/cdd.2009.73

[22] Wang D, et al. Gain-of-Function p53 Mutation Acts as a Genetic Switch for TGFβ Signaling–Induced Epithelial-to-Mesenchymal Transition in Intestinal Tumors. Cancer Res. 2024;84(1):56–68. 37851521 10.1158/0008-5472.CAN-23-1490 PMC10758690

[23] Semenov O, et al. Opposing roles of wild-type and mutant p53 in the process of epithelial to mesenchymal transition. Front Mol Biosci. 2022;9:928399. 35813818 10.3389/fmolb.2022.928399 PMC9261265

[24] Lee S-H, et al. Blocking of p53-Snail binding, promoted by oncogenic K-Ras, recovers p53 expression and function. Neoplasia. 2009;11(1):22–IN6. 19107228 10.1593/neo.81006 PMC2606115

[25] Kalluri R, Weinberg RA. The basics of epithelial-mesenchymal transition. J Clin Investig. 2009;119(6):1420–8. 19487818 10.1172/JCI39104 PMC2689101

[26] Nieto MA. The snail superfamily of zinc-finger transcription factors. Nat Rev Mol Cell Biol. 2002;3(3):155–66. 11994736 10.1038/nrm757

[27] De Craene B, Van Roy F, Berx G. Unraveling signalling cascades for the Snail family of transcription factors. Cell Signal. 2005;17(5):535–47. 15683729 10.1016/j.cellsig.2004.10.011

[28] Cano A, et al. The transcription factor snail controls epithelial–mesenchymal transitions by repressing E-cadherin expression. Nat Cell Biol. 2000;2(2):76–83. 10655586 10.1038/35000025

[29] Navarro P, Lozano E, Cano A. Expression of E-or P-cadherin is not sufficient to modify the morphology and the tumorigenic behavior of murine spindle carcinoma cells: possible involvement of plakoglobin. J Cell Sci. 1993;105(4):923–34. 8227214 10.1242/jcs.105.4.923

[30] Marconi GD, et al. Epithelial-mesenchymal transition (EMT): the type-2 EMT in wound healing, tissue regeneration and organ fibrosis. Cells. 2021;10(7):1587. 34201858 10.3390/cells10071587 PMC8307661

[31] Tomecka P, et al. Factors determining epithelial-mesenchymal transition in cancer progression. Int J Mol Sci. 2024;25(16):8972. 39201656 10.3390/ijms25168972 PMC11354349

[32] Fons JM, Ocaña OH, Nieto MA. Mutual repression between Pax2 and Snail factors regulates the epithelial/mesenchymal state during intermediate mesoderm differentiation. Development. 2025;152(16):dev204848. 40679038 10.1242/dev.204848 PMC12401534

[33] Katsuno Y, Derynck R. Epithelial plasticity, epithelial-mesenchymal transition, and the TGF-β family. Dev Cell. 2021;56(6):726–46. 33756119 10.1016/j.devcel.2021.02.028

[34] Akhund S, Mohammad K. Mechanism of TGFβ in Bone Metastases and its Potential Therapeutic Uses. J Orthop Res Ther. 2023;8:1316.

[35] Lu Z, et al. Partitioning defective 6 homolog alpha (PARD6A) promotes epithelial–mesenchymal transition via integrin β1-ILK-SNAIL1 pathway in ovarian cancer. Cell Death Dis. 2022;13(4):304. 35379775 10.1038/s41419-022-04756-2 PMC8980072

[36] Chattopadhyay I, Ambati R, Gundamaraju R. Exploring the crosstalk between inflammation and epithelial-mesenchymal transition in cancer. Mediat Inflamm. 2021;2021(1):9918379. 10.1155/2021/9918379 PMC8219436 34220337

[37] Baulida J, Díaz VM, García de Herreros A. Snail1: a transcriptional factor controlled at multiple levels. J Clin Med. 2019;8(6):757. 31141910 10.3390/jcm8060757 PMC6616578

[38] Jordà M, et al. Upregulation of MMP-9 in MDCK epithelial cell line in response to expression of the Snail transcription factor. J Cell Sci. 2005;118(15):3371–85. 16079281 10.1242/jcs.02465

[39] Batlle E, et al. The transcription factor snail is a repressor of E-cadherin gene expression in epithelial tumour cells. Nat Cell Biol. 2000;2(2):84–9. 10655587 10.1038/35000034

[40] Müller L, Hatzfeld M, Keil R. Desmosomes as signaling hubs in the regulation of cell behavior. Front cell Dev biology. 2021;9:745670. 10.3389/fcell.2021.745670 PMC8495202 34631720

[41] Guaita S, et al. Snail induction of epithelial to mesenchymal transition in tumor cells is accompanied by MUC1 repression andZEB1 expression. J Biol Chem. 2002;277(42):39209–16. 12161443 10.1074/jbc.M206400200

[42] Kataoka H, et al. A novel Snail-related transcription factor Smuc regulates basic helix–loop–helix transcription factor activities via specific E-box motifs. Nucleic Acids Res. 2000;28(2):626–33. 10606664 10.1093/nar/28.2.626 PMC102498

[43] Kudo-Saito C, et al. Cancer metastasis is accelerated through immunosuppression during Snail-induced EMT of cancer cells. Cancer Cell. 2009;15(3):195–206. 19249678 10.1016/j.ccr.2009.01.023

[44] Jiang M et al. EMT and cancer stem cells: drivers of therapy resistance and promising therapeutic targets. Drug Resist Updates. 2025:101276. 10.1016/j.drup.2025.101276 PMC13560432 40743619

[45] Zhang H, et al. Cancer stem cells, epithelial-mesenchymal transition, ATP and their roles in drug resistance in cancer. Cancer Drug Resist. 2021;4(3):684. 34322664 10.20517/cdr.2021.32 PMC8315560

[46] ёёWang Y, et al. The role of snail in EMT and tumorigenesis. Curr Cancer Drug Targets. 2013;13(9):963–72. 24168186 10.2174/15680096113136660102 PMC4004763

[47] Dzobo K, et al. Advances in therapeutic targeting of cancer stem cells within the tumor microenvironment: an updated review. Cells. 2020;9(8):1896. 32823711 10.3390/cells9081896 PMC7464860

[48] Gillespie MS, Ward CM, Davies CC. DNA repair and therapeutic strategies in cancer stem cells. Cancers. 2023;15(6):1897. 36980782 10.3390/cancers15061897 PMC10047301

[49] Qin Q, et al. Targeting the EMT transcription factor Snail overcomes resistance to osimertinib in EGFR-mutant non‐small cell lung cancer. Thorac Cancer. 2021;12(11):1708–15. 33943009 10.1111/1759-7714.13906 PMC8169301

[50] Da Fonseca LM, et al. Glycosylation in cancer: interplay between multidrug resistance and epithelial-to-mesenchymal transition? Front Oncol. 2016;6:p158. 10.3389/fonc.2016.00158 PMC4916178 27446804

[51] Seo J, et al. The role of epithelial–mesenchymal transition-regulating transcription factors in anti-cancer drug resistance. Arch Pharm Res. 2021;44(3):281–92. 33768509 10.1007/s12272-021-01321-x PMC8009775

[52] Liang H, et al. Snail expression contributes to temozolomide resistance in glioblastoma. Am J Translational Res. 2019;11(7):4277. PMC6684932 31396334

[53] Wang H, et al. Regulation of ATP-binding cassette subfamily B member 1 by snail contributes to chemoresistance in colorectal cancer. Cancer Sci. 2020;111(1):84–97. 31774615 10.1111/cas.14253 PMC6942434

[54] Liu F, et al. miR-153 enhances the therapeutic effect of gemcitabine by targeting Snail in pancreatic cancer. Acta Biochim Biophys Sin. 2017;49(6):520–9. 28459992 10.1093/abbs/gmx039

[55] Assani G, Zhou Y. Effect of modulation of epithelial-mesenchymal transition regulators Snail1 and Snail2 on cancer cell radiosensitivity by targeting of the cell cycle, cell apoptosis and cell migration/invasion. Oncol Lett. 2019;17(1):23–30. 30655734 10.3892/ol.2018.9636 PMC6313178

[56] Wang H et al. Silencing snail reverses epithelial-mesenchymal transition and increases radiosensitivity in hypopharyngeal carcinoma. OncoTargets therapy. 2020:497–511. 10.2147/OTT.S237410 PMC6970617 32021293

[57] Escriva M, et al. Repression of PTEN phosphatase by Snail1 transcriptional factor during gamma radiation-induced apoptosis. Mol Cell Biol. 2008;28(5):1528–40. 18172008 10.1128/MCB.02061-07 PMC2258777

[58] Vega S, et al. Snail blocks the cell cycle and confers resistance to cell death. Genes Dev. 2004;18(10):1131–43. 15155580 10.1101/gad.294104 PMC415638

[59] Faget J, et al. Neutrophils and snail orchestrate the establishment of a pro-tumor microenvironment in lung cancer. Cell Rep. 2017;21(11):3190–204. 29241546 10.1016/j.celrep.2017.11.052

[60] Taki M, et al. Snail promotes ovarian cancer progression by recruiting myeloid-derived suppressor cells via CXCR2 ligand upregulation. Nat Commun. 2018;9(1):1685. 29703902 10.1038/s41467-018-03966-7 PMC5923228

[61] Kudo-Saito C, et al. CCL2 is critical for immunosuppression to promote cancer metastasis. Clin Exp Metastasis. 2013;30(4):393–405. 23143679 10.1007/s10585-012-9545-6

[62] Dongre A, et al. Direct and indirect regulators of epithelial–mesenchymal transition–mediated immunosuppression in breast carcinomas. Cancer Discov. 2021;11(5):1286–305. 33328216 10.1158/2159-8290.CD-20-0603 PMC8432413

[63] Dong B, Wu Y. Epigenetic regulation and post-translational modifications of SNAI1 in cancer metastasis. Int J Mol Sci. 2021;22(20):11062. 34681726 10.3390/ijms222011062 PMC8538584

[64] Xie Y, et al. Gastrointestinal cancers in China, the USA, and Europe. Gastroenterol Rep. 2021;9(2):91–104. 10.1093/gastro/goab010 PMC8128023 34026216

[65] Yastrebova MA, et al. Snail-family proteins: role in carcinogenesis and prospects for antitumor therapy. Acta naturae. 2021;13(1):76. 33959388 10.32607/actanaturae.11062 PMC8084295

[66] Brzozowa M, et al. The role of Snail1 transcription factor in colorectal cancer progression and metastasis. Contemp Oncology/Współczesna Onkologia. 2015;19(4):265–70. 10.5114/wo.2014.42173 PMC4631295 26557772

[67] Zheng H, et al. Glycogen synthase kinase-3 beta regulates Snail and β-catenin expression during Fas-induced epithelial–mesenchymal transition in gastrointestinal cancer. Eur J Cancer. 2013;49(12):2734–46. 23582741 10.1016/j.ejca.2013.03.014

[68] Xu X, et al. Hyperglycemia promotes Snail-induced epithelial–mesenchymal transition of gastric cancer via activating ENO1 expression. Cancer Cell Int. 2019;19(1):344. 31889896 10.1186/s12935-019-1075-8 PMC6924061

[69] Yu J, et al. Snail enhances glycolysis in the epithelial-mesenchymal transition process by targeting FBP1 in gastric cancer. Cell Physiol Biochem. 2017;43(1):31–8. 28848200 10.1159/000480314

[70] Chen R, et al. SNAIL regulates gastric carcinogenesis through CCN3 and NEFL. Carcinogenesis. 2021;42(2):190–201. 33313663 10.1093/carcin/bgaa133

[71] Bao Z, et al. SNAIL induces EMT and lung metastasis of tumours secreting CXCL2 to promote the invasion of M2-type immunosuppressed macrophages in colorectal cancer. Int J Biol Sci. 2022;18(7):2867. 35541899 10.7150/ijbs.66854 PMC9066124

[72] Hwang WL, et al. SNAIL regulates interleukin-8 expression, stem cell–like activity, and tumorigenicity of human colorectal carcinoma cells. Gastroenterology. 2011;141(1):279–91. e5. 21640118 10.1053/j.gastro.2011.04.008

[73] Conciatori F, et al. Colorectal cancer stem cells properties and features: Evidence of interleukin-8 involvement. Cancer Drug Resist. 2019;2(4):968. 35582268 10.20517/cdr.2019.56 PMC9019202

[74] Leng Z, et al. Krüppel-like factor 4 regulates stemness and mesenchymal properties of colorectal cancer stem cells through the TGF‐β1/Smad/snail pathway. J Cell Mol Med. 2020;24(2):1866–77. 31830379 10.1111/jcmm.14882 PMC6991673

[75] Jing C, et al. The PSMD14 inhibitor Thiolutin as a novel therapeutic approach for esophageal squamous cell carcinoma through facilitating SNAIL degradation. Theranostics. 2021;11(12):5847. 33897885 10.7150/thno.46109 PMC8058732

[76] Lv X-L, et al. Snail family transcriptional repressor 1 radiosensitizes esophageal cancer via epithelial-mesenchymal transition signaling: From bioinformatics to integrated study. World J Gastrointest Oncol. 2025;17(4):97644. 40235866 10.4251/wjgo.v17.i4.97644 PMC11995309

[77] Kowal A, et al. Human leukocyte antigen (HLA)-G gene polymorphism in patients with non‐small cell lung cancer. Thorac cancer. 2015;6(5):613–9. 26445610 10.1111/1759-7714.12232 PMC4567007

[78] Müller-Hermelink HK, et al. Pathology & genetics, tumours of the lung, pleura, thymus and heart. In: Travis WD, Brambilla E, Müller-Hermelink HK, Harris CC, editors. World Health Organization Classification of Tumors. Lyon: IARC; 2004. pp. 146–7.

[79] Xue Q, et al. LASP1 Induces Epithelial-Mesenchymal Transition in Lung Cancer through the TGF‐β1/Smad/Snail Pathway. Can Respir J. 2021;2021(1):5277409. 34912481 10.1155/2021/5277409 PMC8668282

[80] Liu J, et al. Rhein inhibits the progression of chemoresistant lung cancer cell lines via the Stat3/Snail/MMP2/MMP9 pathway. Biomed Res Int. 2022;2022(1):7184871. 35178453 10.1155/2022/7184871 PMC8846980

[81] Liu W et al. RIPK2/STAT3 signaling axis drives lung cancer metastasis through SNAIL activation: molecular mechanisms and clinical implications. Cell Signal. 2025:112211. 10.1016/j.cellsig.2025.112211 41192526

[82] Yanagawa J, et al. Snail promotes CXCR2 liganddependent tumor progression in nonsmall cell lung carcinoma. Clin Cancer Res. 2009;15(22):6820–9. 19887480 10.1158/1078-0432.CCR-09-1558 PMC2783274

[83] Cheng H-Y, et al. Snail-regulated exosomal microRNA-21 suppresses NLRP3 inflammasome activity to enhance cisplatin resistance. J Immunother Cancer. 2022;10(8):e004832. 36002186 10.1136/jitc-2022-004832 PMC9413180

[84] Liu C-W, et al. Snail regulates Nanog status during the epithelial–mesenchymal transition via the Smad1/Akt/GSK3β signaling pathway in non-small-cell lung cancer. Oncotarget. 2014;5(11):3880. 25003810 10.18632/oncotarget.2006 PMC4116528

[85] Zhu K, et al. MET inhibitor, capmatinib overcomes osimertinib resistance via suppression of MET/Akt/snail signaling in non-small cell lung cancer and decreased generation of cancer-associated fibroblasts. Aging. 2021;13(5):6890. 33621951 10.18632/aging.202547 PMC7993678

[86] Battifora H, McCaughey WE. Tumors of the serosal membranes. American Registry of Pathology; 1995.

[87] Robinson BW, Musk AW, Lake RA. Malignant mesothelioma. Lancet. 2005;366(9483):397–408. 16054941 10.1016/S0140-6736(05)67025-0

[88] Cho J-H, et al. NF2 blocks Snail-mediated p53 suppression in mesothelioma. Oncotarget. 2015;6(12):10073. 25823924 10.18632/oncotarget.3543 PMC4496341

[89] Schelch K, et al. YB-1 regulates mesothelioma cell migration via snail but not EGFR, MMP1, EPHA5 or PARK2. Mol Oncol. 2024;18(4):815–31. 36550787 10.1002/1878-0261.13367 PMC10994239

[90] Hsieh JJ, et al. Renal cell carcinoma. Nat reviews Disease primers. 2017;3(1):1–19. 10.1038/nrdp.2017.9 PMC5936048 28276433

[91] Mikami S, et al. Expression of Snail and Slug in renal cell carcinoma: E-cadherin repressor Snail is associated with cancer invasion and prognosis. Lab Invest. 2011;91(10):1443–58. 21808237 10.1038/labinvest.2011.111

[92] Liu Y, et al. Cyclovirobuxine inhibits the progression of clear cell renal cell carcinoma by suppressing the IGFBP3-AKT/STAT3/MAPK-Snail signalling pathway. Int J Biol Sci. 2021;17(13):3522. 34512163 10.7150/ijbs.62114 PMC8416721

[93] Lin TC, et al. Ghrelin promotes renal cell carcinoma metastasis via Snail activation and is associated with poor prognosis. J Pathol. 2015;237(1):50–61. 25925728 10.1002/path.4552

[94] Bralten LB, French PJ. Genetic alterations in glioma. Cancers. 2011;3(1):1129–40. 24212656 10.3390/cancers3011129 PMC3756406

[95] Lathia JD, et al. Cancer stem cells in glioblastoma. Genes Dev. 2015;29(12):1203–17. 26109046 10.1101/gad.261982.115 PMC4495393

[96] Mahabir R, et al. Sustained elevation of Snail promotes glial-mesenchymal transition after irradiation in malignant glioma. Neurooncology. 2014;16(5):671–85. 10.1093/neuonc/not239 PMC3984547 24357458

[97] 명재경. Snail and ZEB2 play an oncogenic role in pediatric and adult glioblastoma cells through the induction of epithelial mesenchymal transition like process, 2015, 서울대학교 대학&#50896.

[98] Yuan Y et al. LKB1 suppresses glioma cell invasion via NF-κB/Snail signaling repression. OncoTargets Therapy. 2019:2451–63. 10.2147/OTT.S193736 PMC6452796 31040689

[99] Wang N, et al. Overexpression of FBXO17 promotes the proliferation, migration and invasion of glioma cells through the Akt/GSK-3β/snail pathway. Cell Transplant. 2021;30:09636897211007395. 33853342 10.1177/09636897211007395 PMC8058804

[100] Hardy RG, et al. Snail family transcription factors are implicated in thyroid carcinogenesis. Am J Pathol. 2007;171(3):1037–46. 17724139 10.2353/ajpath.2007.061211 PMC1959496

[101] Wang N, et al. Expression of TGF-β1, SNAI1 and MMP-9 is associated with lymph node metastasis in papillary thyroid carcinoma. J Mol Histol. 2014;45(4):391–9. 24276590 10.1007/s10735-013-9557-9

[102] Wieczorek-Szukala K, et al. Snail-1 overexpression correlates with metastatic phenotype in BRAFV600E positive papillary thyroid carcinoma. J Clin Med. 2020;9(9):2701. 32825554 10.3390/jcm9092701 PMC7565998

[103] Johnson DE, et al. Head and neck squamous cell carcinoma. Nat reviews Disease primers. 2020;6(1):92. 33243986 10.1038/s41572-020-00224-3 PMC7944998

[104] Yang S, et al. STC2 promotes head and neck squamous cell carcinoma metastasis through modulating the PI3K/AKT/snail signaling. Oncotarget. 2017;8:5976–91. 27863406 10.18632/oncotarget.13355 PMC5351606

[105] Nieh S, et al. Regulation of tumor progression via the Snail-RKIP signaling pathway by nicotine exposure in head and neck squamous cell carcinoma. Head Neck. 2015;37(12):1712–21. 24986226 10.1002/hed.23820

[106] Dennis M, et al. Snail controls the mesenchymal phenotype and drives erlotinib resistance in oral epithelial and head and neck squamous cell carcinoma cells. Otolaryngology–Head Neck Surg. 2012;147(4):726–32. 10.1177/0194599812446407 PMC4167686 22568942

[107] Ryu K-J, et al. Chaperone-mediated autophagy modulates Snail protein stability: implications for breast cancer metastasis. Mol Cancer. 2024;23(1):227. 39390584 10.1186/s12943-024-02138-0 PMC11468019

[108] Xu M, et al. SNAI1 promotes the cholangiocellular phenotype, but not epithelial–mesenchymal transition, in a murine hepatocellular carcinoma model. Cancer Res. 2019;79(21):5563–74. 31383647 10.1158/0008-5472.CAN-18-3750 PMC7237201

[109] Paul MC, et al. Non-canonical functions of SNAIL drive context-specific cancer progression. Nat Commun. 2023;14(1):1201. 36882420 10.1038/s41467-023-36505-0 PMC9992512

[110] Zheng X, et al. Epithelial-to-mesenchymal transition is dispensable for metastasis but induces chemoresistance in pancreatic cancer. Nature. 2015;527(7579):525–30. 26560028 10.1038/nature16064 PMC4849281

[111] Krebs AM, et al. The EMT-activator Zeb1 is a key factor for cell plasticity and promotes metastasis in pancreatic cancer. Nat Cell Biol. 2017;19(5):518–29. 28414315 10.1038/ncb3513

[112] Wang G, et al. Prognostic value of Twist, Snail and E-cadherin expression in pathological N0 non-small-cell lung cancer: a retrospective cohort study. Eur J Cardiothorac Surg. 2018;54(2):237–45. 29415155 10.1093/ejcts/ezy022

[113] Brzozowa-Zasada M. Immunohistochemical expression of Snail1 protein in colorectal adenocarcinoma samples and its prognostic activity in Caucasian patients. Gastroenterol Review/Przegląd Gastroenterologiczny. 2021;16(4):339–45. 10.5114/pg.2021.111765 PMC8690945 34976242

[114] He H, et al. Snail is an independent prognostic predictor for progression and patient survival of gastric cancer. Cancer Sci. 2012;103(7):1296–303. 22471696 10.1111/j.1349-7006.2012.02295.x PMC7659386

[115] Jouppila-Mättö A, et al. Twist and snai1 expression in pharyngeal squamous cell carcinoma stroma is related to cancer progression. BMC Cancer. 2011;11(1):350. 21834956 10.1186/1471-2407-11-350 PMC3173446

[116] Elbe P, et al. Pharyngeal squamous cell carcinoma and risk of later esophageal squamous cell carcinoma–A nationwide population-based matched case-control study. Cancer Epidemiol. 2025;98:102876. 40669223 10.1016/j.canep.2025.102876

[117] Blechschmidt K, et al. The E-cadherin repressor Snail is associated with lower overall survival of ovarian cancer patients. Br J Cancer. 2008;98(2):489–95. 18026186 10.1038/sj.bjc.6604115 PMC2361433

[118] Kong D, et al. Prognostic significance of snail expression in hilar cholangiocarcinoma. Braz J Med Biol Res. 2012;45:617–24. 22570087 10.1590/S0100-879X2012007500070 PMC3854268

[119] Zivotic M, et al. SLUG and SNAIL as potential immunohistochemical biomarkers for renal cancer staging and survival. Int J Mol Sci. 2023;24(15):12245. 37569620 10.3390/ijms241512245 PMC10418944

[120] Wu W-S, et al. Snail collaborates with EGR-1 and SP-1 to directly activate transcription of MMP 9 and ZEB1. Sci Rep. 2017;7(1):17753. 29259250 10.1038/s41598-017-18101-7 PMC5736704

[121] Ikenouchi J, et al. Regulation of tight junctions during the epithelium-mesenchyme transition: direct repression of the gene expression of claudins/occludin by Snail. J Cell Sci. 2003;116(10):1959–67. 12668723 10.1242/jcs.00389

[122] Stanisavljevic J, et al. The p65 subunit of NF-κB and PARP1 assist Snail1 in activating fibronectin transcription. J Cell Sci. 2011;124(24):4161–71. 22223884 10.1242/jcs.078824

[123] Liu J, et al. Collagen 1A1 (COL1A1) promotes metastasis of breast cancer and is a potential therapeutic target. Discov Med. 2018;25(139):211–23. 29906404

[124] Sundararajan V, et al. SNAI1 recruits HDAC1 to suppress SNAI2 transcription during epithelial to mesenchymal transition. Sci Rep. 2019;9(1):8295. 31165775 10.1038/s41598-019-44826-8 PMC6549180

[125] Kim NH, et al. Snail reprograms glucose metabolism by repressing phosphofructokinase PFKP allowing cancer cell survival under metabolic stress. Nat Commun. 2017;8(1):14374. 28176759 10.1038/ncomms14374 PMC5309788

[126] Beach S, et al. Snail is a repressor of RKIP transcription in metastatic prostate cancer cells. Oncogene. 2008;27(15):2243–8. 17952120 10.1038/sj.onc.1210860 PMC2933472

[127] Kashyap A, et al. The human Lgl polarity gene, Hugl-2, induces MET and suppresses Snail tumorigenesis. Oncogene. 2013;32(11):1396–407. 22580609 10.1038/onc.2012.162

[128] Massoumi R, et al. Down-regulation of CYLD expression by Snail promotes tumor progression in malignant melanoma. J Exp Med. 2009;206(1):221–32. 19124656 10.1084/jem.20082044 PMC2626666

[129] Whiteman E, et al. The transcription factor snail represses Crumbs3 expression and disrupts apico-basal polarity complexes. Oncogene. 2008;27(27):3875–9. 18246119 10.1038/onc.2008.9 PMC2533733

[130] Ly TM, et al. Snail upregulates transcription of FN, LEF, COX2, and COL1A1 in hepatocellular carcinoma: a general model established for snail to transactivate mesenchymal genes. Cells. 2021;10(9):2202. 34571852 10.3390/cells10092202 PMC8467536

[131] Neal CL, et al. Snail transcription factor negatively regulates maspin tumor suppressor in human prostate cancer cells. BMC Cancer. 2012;12(1):336. 22857708 10.1186/1471-2407-12-336 PMC3437215

[132] Ly TM et al. Snail upregulates FN and LEF transcription negatively feed backed by PPAR-gamma: a general model established for Snail to transactivate mesenchymal genes. 2021. 10.3390/cells10092202 PMC8467536 34571852

[133] Dong C, et al. Loss of FBP1 by Snail-mediated repression provides metabolic advantages in basal-like breast cancer. Cancer Cell. 2013;23(3):316–31. 23453623 10.1016/j.ccr.2013.01.022 PMC3703516

[134] Wang J, et al. Snail determines the therapeutic response to mTOR kinase inhibitors by transcriptional repression of 4E-BP1. Nat Commun. 2017;8(1):2207. 29263324 10.1038/s41467-017-02243-3 PMC5738350

[135] Rönsch K, et al. SNAIL1 combines competitive displacement of ASCL2 and epigenetic mechanisms to rapidly silence the EPHB3 tumor suppressor in colorectal cancer. Mol Oncol. 2015;9(2):335–54. 25277775 10.1016/j.molonc.2014.08.016 PMC5528665

[136] Vincent T, et al. A SNAIL1–SMAD3/4 transcriptional repressor complex promotes TGF-β mediated epithelial–mesenchymal transition. Nat Cell Biol. 2009;11(8):943–50. 19597490 10.1038/ncb1905 PMC3769970

[137] Pálmer HG, et al. The transcription factor SNAIL represses vitamin D receptor expression and responsiveness in human colon cancer. Nat Med. 2004;10(9):917–9. 15322538 10.1038/nm1095

[138] Kajita M, McClinic KN, Wade PA. Aberrant expression of the transcription factors snail and slug alters the response to genotoxic stress. Mol Cell Biol. 2004;24(17):7559–66. 15314165 10.1128/MCB.24.17.7559-7566.2004 PMC506998

[139] Grotegut S, et al. Hepatocyte growth factor induces cell scattering through MAPK/Egr-1‐mediated upregulation of Snail. EMBO J. 2006;25(15):3534–45. 16858414 10.1038/sj.emboj.7601213 PMC1538570

[140] Dhasarathy A, Kajita M, Wade PA. The transcription factor snail mediates epithelial to mesenchymal transitions by repression of estrogen receptor-α. Mol Endocrinol. 2007;21(12):2907–18. 17761946 10.1210/me.2007-0293 PMC2668600

[141] Reinke LM, Xu Y, Cheng C. Snail represses the splicing regulator epithelial splicing regulatory protein 1 to promote epithelial-mesenchymal transition. J Biol Chem. 2012;287(43):36435–42. 22961986 10.1074/jbc.M112.397125 PMC3476309

[142] De Craene B, et al. The transcription factor snail induces tumor cell invasion through modulation of the epithelial cell differentiation program. Cancer Res. 2005;65(14):6237–44. 16024625 10.1158/0008-5472.CAN-04-3545

[143] Xu Z, et al. Snail1-dependent transcriptional repression of Cezanne2 in hepatocellular carcinoma. Oncogene. 2014;33(22):2836–45. 23792447 10.1038/onc.2013.243

[144] Fazilaty H, et al. A gene regulatory network to control EMT programs in development and disease. Nat Commun. 2019;10(1):5115. 31712603 10.1038/s41467-019-13091-8 PMC6848104

[145] Kao Y-C, et al. Downregulation of thrombomodulin, a novel target of Snail, induces tumorigenesis through epithelial-mesenchymal transition. Mol Cell Biol. 2010;30(20):4767–85. 20713448 10.1128/MCB.01021-09 PMC2950553

[146] Chang C-J, et al. p53 regulates epithelial–mesenchymal transition and stem cell properties through modulating miRNAs. Nat Cell Biol. 2011;13(3):317–23. 21336307 10.1038/ncb2173 PMC3075845

[147] Shuvalov O, et al. Linking metabolic reprogramming, plasticity and tumor progression. Cancers. 2021;13(4):762. 33673109 10.3390/cancers13040762 PMC7917602

[148] Wang Y, et al. Critical role for transcriptional repressor Snail2 in transformation by oncogenic RAS in colorectal carcinoma cells. Oncogene. 2010;29(33):4658–70. 20562906 10.1038/onc.2010.218 PMC7646260

[149] Horiguchi K, et al. Role of Ras signaling in the induction of snail by transforming growth factor-β. J Biol Chem. 2009;284(1):245–53. 19010789 10.1074/jbc.M804777200

[150] Han JH, et al. Snail acetylation by autophagy-derived acetyl‐coenzyme A promotes invasion and metastasis of KRAS‐LKB1 co‐mutated lung cancer cells. Cancer Commun. 2022;42(8):716–49. 10.1002/cac2.12332 PMC9395322 35838183

[151] Mittenberg AG, Moiseeva TN, Barlev NA. Role of proteasomes in transcription and their regulation by covalent modifications. Front Biosci. 2008;13:7184–92. 18508726 10.2741/3220

[152] Wang B, et al. The role of the transcription factor EGR1 in cancer. Front Oncol. 2021;11:642547. 33842351 10.3389/fonc.2021.642547 PMC8024650

[153] Beishline K, Azizkhan-Clifford J. Sp1 and the ‘hallmarks of cancer’. FEBS J. 2015;282(2):224–58. 25393971 10.1111/febs.13148

[154] Rembold M, et al. A conserved role for Snail as a potentiator of active transcription. Genes Dev. 2014;28(2):167–81. 24402316 10.1101/gad.230953.113 PMC3909790

[155] Vistain LF, et al. Targeted Inhibition of Snail Activity in Breast Cancer Cells by Using a CoIII-Ebox Conjugate. ChemBioChem. 2015;16(14):2065–72. 26305708 10.1002/cbic.201500289 PMC4638217

[156] Leyssens L, et al. Cobalt toxicity in humans—A review of the potential sources and systemic health effects. Toxicology. 2017;387:43–56. 28572025 10.1016/j.tox.2017.05.015

[157] Lee S, et al. Antitumor effect of novel small chemical inhibitors of Snail-p53 binding in K-Ras-mutated cancer cells. Oncogene. 2010;29(32):4576–87. 20531295 10.1038/onc.2010.208

[158] Azmi AS, et al. Systems analysis reveals a transcriptional reversal of the mesenchymal phenotype induced by SNAIL-inhibitor GN-25. BMC Syst Biol. 2013;7(1):85. 24004452 10.1186/1752-0509-7-85 PMC3848843

[159] Cui H, et al. Design and synthesis of dual inhibitors targeting snail and histone deacetylase for the treatment of solid tumour cancer. Eur J Med Chem. 2022;229:114082. 34995925 10.1016/j.ejmech.2021.114082

[160] Li H-M, et al. A potent CBP/p300-Snail interaction inhibitor suppresses tumor growth and metastasis in wild-type p53-expressing cancer. Sci Adv. 2020;6(17):eaaw8500. 32494626 10.1126/sciadv.aaw8500 PMC7176418

[161] Ferrari-Amorotti G, et al. Inhibiting interactions of lysine demethylase LSD1 with snail/slug blocks cancer cell invasion. Cancer Res. 2013;73(1):235–45. 23054398 10.1158/0008-5472.CAN-12-1739 PMC3537890

[162] Shah P, Gau Y, Sabnis G. Histone deacetylase inhibitor entinostat reverses epithelial to mesenchymal transition of breast cancer cells by reversing the repression of E-cadherin. Breast Cancer Res Treat. 2014;143(1):99–111. 24305977 10.1007/s10549-013-2784-7

[163] Morgunkova A, Barlev NA. Lysine methylation goes global. Cell Cycle. 2006;5(12):1308–12. 16760670 10.4161/cc.5.12.2820

[164] O’BRIEN S, et al. Blood pressure effects of tranylcypromine when prescribed singly and in combination with amitriptyline. J Clin Psychopharmacol. 1992;12(2):104–9. 1573032

[165] Weber-Grandke H, et al. The pharmacokinetics of tranylcypromine enantiomers in healthy subjects after oral administration of racemic drug and the single enantiomers. Br J Clin Pharmacol. 1993;36(4):363–5. 12959316 10.1111/j.1365-2125.1993.tb00377.x PMC1364691

[166] Mallinger AG, et al. Pharmacokinetics of tranylcypromine in patients who are depressed: relationship to cardiovascular effects. Clin Pharmacol Ther. 1986;40(4):444–50. 3757407 10.1038/clpt.1986.205

[167] Ulrich S, Ricken R, Adli M. Tranylcypromine in mind (Part I): Review of pharmacology. Eur Neuropsychopharmacol. 2017;27(8):697–713. 28655495 10.1016/j.euroneuro.2017.05.007

[168] Salamero García O, et al. First-in-human phase I study of iadademstat (ORY-1001): a first-in-class lysine-specific histone demethylase 1A inhibitor in relapsed or refractory acute myeloid leukemia. J Clin Oncol. 2020. (American Society of Clinical Oncology). 10.1200/JCO.19.03250 PMC7768337 33052756

[169] Goethert JR, et al. Bomedemstat (IMG-7289), an LSD1 inhibitor, manages the signs and symptoms of essential thrombocythemia (ET) while reducing the burden of cells homozygous for driver mutations. Blood. 2023;142:747.

[170] Johnston G, et al. Nascent transcript and single-cell RNA-seq analysis defines the mechanism of action of the LSD1 inhibitor INCB059872 in myeloid leukemia. Gene. 2020;752:144758. 32422235 10.1016/j.gene.2020.144758 PMC7401316

[171] Hollebecque A, et al. Clinical activity of CC-90011, an oral, potent, and reversible LSD1 inhibitor, in advanced malignancies. Cancer. 2022;128(17):3185–95. 35737639 10.1002/cncr.34366 PMC9540525

[172] Connolly RM, Rudek MA, Piekarz R. Entinostat: a promising treatment option for patients with advanced breast cancer. Future Oncol. 2017;13(13):1137–48. 28326839 10.2217/fon-2016-0526 PMC5618943

[173] Peinado H, et al. Snail mediates E-cadherin repression by the recruitment of the Sin3A/histone deacetylase 1 (HDAC1)/HDAC2 complex. Mol Cell Biol. 2004;24(1):306–19. 14673164 10.1128/MCB.24.1.306-319.2004 PMC303344

[174] Javaid S, et al. Dynamic chromatin modification sustains epithelial-mesenchymal transition following inducible expression of Snail-1. Cell Rep. 2013;5(6):1679–89. 24360956 10.1016/j.celrep.2013.11.034 PMC4034764

[175] Shen P-C, Chang P-C, Hsieh J-L. Snail regulation in fibroblast-like synoviocytes by a histone deacetylase or glycogen synthase kinase inhibitor affects cell proliferation and gene expression. PLoS ONE. 2021;16(9):e0257839. 34582486 10.1371/journal.pone.0257839 PMC8478242

[176] Tomono T, et al. Entinostat reverses P-glycoprotein activation in snail-overexpressing adenocarcinoma HCC827 cells. PLoS ONE. 2018;13(7):e0200015. 29979729 10.1371/journal.pone.0200015 PMC6034804

[177] Baretti M, et al. Entinostat in combination with nivolumab in metastatic pancreatic ductal adenocarcinoma: a phase 2 clinical trial. Nat Commun. 2024;15(1):9801. 39532835 10.1038/s41467-024-52528-7 PMC11557583

[178] Connolly RM, et al. E2112: randomized phase III trial of endocrine therapy plus entinostat or placebo in hormone receptor–positive advanced breast cancer. A trial of the ECOG-ACRIN cancer research group. J Clin Oncol. 2021;39(28):3171–81. 34357781 10.1200/JCO.21.00944 PMC8478386

[179] Wang J, et al. Phase I study and pilot efficacy analysis of entinostat, a novel histone deacetylase inhibitor, in chinese postmenopausal women with hormone receptor-positive metastatic breast cancer. Target Oncol. 2021;16(5):591–9. 34196874 10.1007/s11523-021-00823-4 PMC8484140

[180] Tomita Y, et al. The interplay of epigenetic therapy and immunity in locally recurrent or metastatic estrogen receptor-positive breast cancer: correlative analysis of ENCORE 301, a randomized, placebo-controlled phase II trial of exemestane with or without entinostat. Oncoimmunology. 2016;5(11):e1219008. 27999738 10.1080/2162402X.2016.1219008 PMC5139687

[181] Ryan QC, et al. Phase I and pharmacokinetic study of MS-275, a histone deacetylase inhibitor, in patients with advanced and refractory solid tumors or lymphoma. J Clin Oncol. 2005;23(17):3912–22. 15851766 10.1200/JCO.2005.02.188

[182] Pili R, et al. Phase I study of the histone deacetylase inhibitor entinostat in combination with 13-cis retinoic acid in patients with solid tumours. Br J Cancer. 2012;106(1):77–84. 22134508 10.1038/bjc.2011.527 PMC3251867

[183] Herranz N, et al. Polycomb complex 2 is required for E-cadherin repression by the Snail1 transcription factor. Mol Cell Biol. 2008;28(15):4772–81. 18519590 10.1128/MCB.00323-08 PMC2493371

[184] Battistelli C, et al. The Snail repressor recruits EZH2 to specific genomic sites through the enrollment of the lncRNA HOTAIR in epithelial-to-mesenchymal transition. Oncogene. 2017;36(7):942–55. 27452518 10.1038/onc.2016.260 PMC5318668

