# Supplementary material for: Decoding the Snail transcriptional network: its role in cancer progression and therapy
Source: Biol Direct. 2026 May 18;21:69. doi: 10.1186/s13062-026-00751-1 (PMC13181986; doi:10.1186/s13062-026-00751-1)
Supplement: Supplementary file 1 — Supplementary Material 1: Supplementary Table 1. P-values for gene expression correlations. Link to Fig. 3. [file 13062_2026_751_MOESM1_ESM.docx]

**Supplement**

**Methods**

Python 3.11 was used to obtain and analyze the data.

The SNAI1 gene expression and survival data were obtained from the GDC database at https://portal.gdc.cancer.gov/. The data were obtained using the GDC Application Programming Interface (GDC API). The lifelines library's KaplanMeierFitter class and the matplotlib.pyplot module were used to plot the Kaplan-Meier curves. The logrank_test function of the Statistics module in Lifelines was used to calculate the p-value.

Correlation analysis of SNAI1 expression and target genes was performed using the GDC database. Gene expression was measured in UQFPKM units and then transformed to log2(UQFPKM + 1). The pearsonr function in the scipy.stats module was used to calculate the Pearson correlation coefficient (r) and p-value.

To search for Snail binding sites with target genes, we obtained the coordinates of the gene and regulatory element sequences, the gene sequences, and the upstream sequences using the Ensembl REST API (https://rest.ensembl.org/). The frequency matrix of the SNAI1 transcription factor binding motif was obtained from the JASPAR 2024 database (https://jaspar.elixir.no/), matrix id MA1558.2. The FIMO MEME tool (https://meme-suite.org/meme/tools/fimo) was used to search for SNAI1 binding sites with target gene sequences using the given Jasp(CAGGTG) motif frequency matrix at a P-value of 0.0001. The Jasp(CAGGTG) E-box search was performed only in promoter and enhancer sequences located within the range of -10,000 to +60,000 bp downstream of the transcription start site (TSS). The Biopython SeqIO program was used to search for CAGGTG and CANNTG motifs in the promoter sequences of target genes.
