# Supplementary figures and images for: Decoding the Snail transcriptional network: its role in cancer progression and therapy
Source: Biol Direct. 2026 May 18;21:69. doi: 10.1186/s13062-026-00751-1 (PMC13181986; doi:10.1186/s13062-026-00751-1)

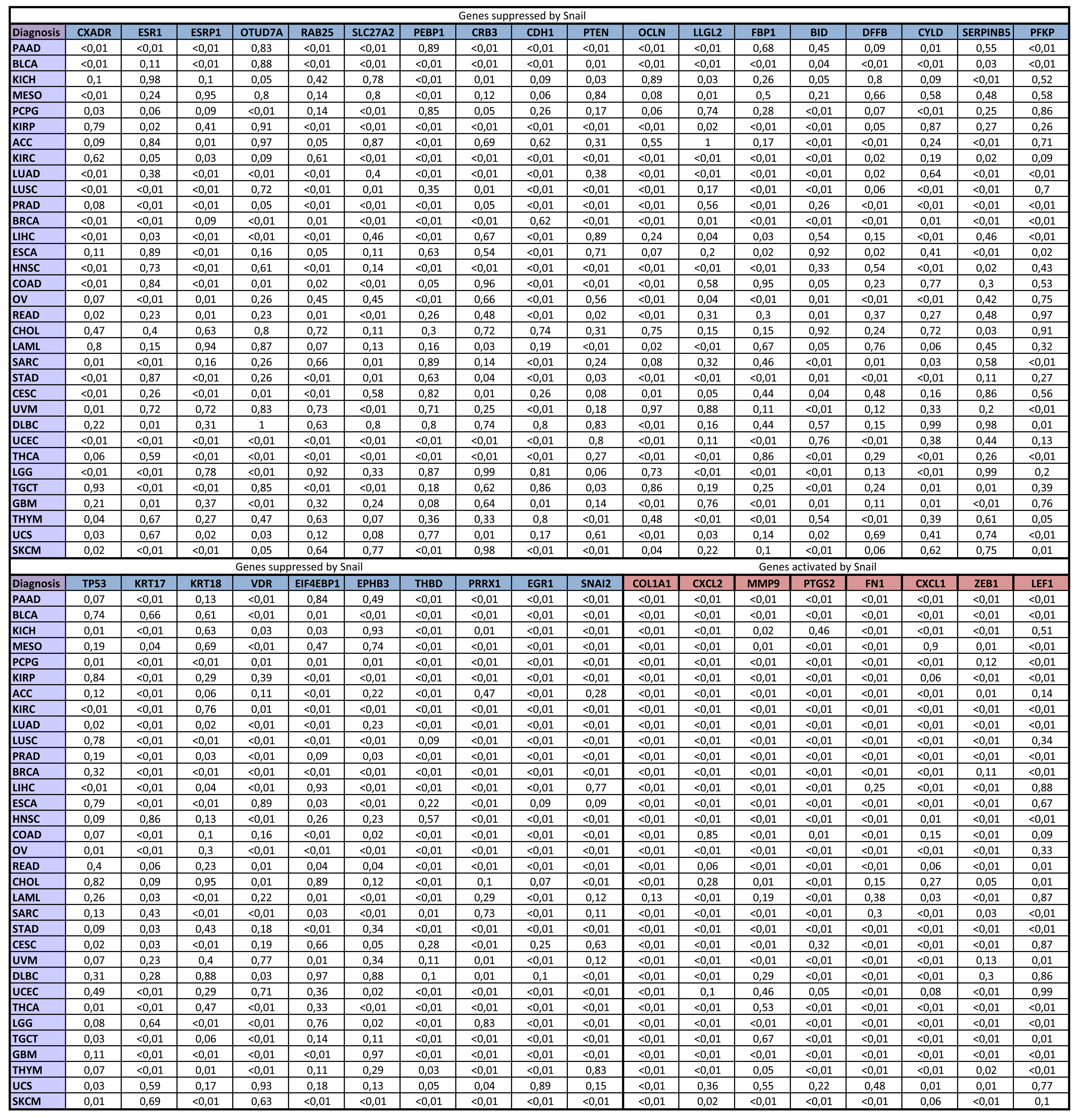

Supplement: Supplementary file 2 — Supplementary Material 2: Additional file 1. Methods.docx. The description of bioinformatics and statistical methods used. [file 13062_2026_751_MOESM2_ESM.jpg]
